# Crystal structure, interaction energies and experimental electron density of the popular drug ketoprophen

**DOI:** 10.1107/S2052252518013222

**Published:** 2018-10-27

**Authors:** Sylwia Pawlędzio, Anna Makal, Damian Trzybiński, Krzysztof Woźniak

**Affiliations:** a Biological and Chemical Research Centre, Department of Chemistry, University of Warsaw, Żwirki i Wigury 101, Warsaw 02-089, Poland

**Keywords:** ketoprophen, charge density, crystal structures, pharmaceuticals, bioinversion

## Abstract

The crystal and molecular structure of the pure (*S*)-enantiomer of the popular analgesic and anti-inflammatory drug ketoprophen (α-ket) is reported. A detailed aspherical charge-density model based on high-resolution X-ray diffraction data has been refined, yielding a high-precision geometric description and classification of the O—H⋯O interactions as medium strength hydrogen bonds. The crystal structure of the racemic form of ketoprophen (β-ket) was also redetermined at 100 K, at 0.5 Å resolution. A previously unreported disorder (10% occupancy) was discovered. In contrast to the racemic β-ket case, the (*S*)-enantiomer crystallizes with two independent molecules in the asymmetric unit with two distinct conformations. The major difference between the β-ket and α-ket crystal forms lies in the formation of distinct hydrogen-bonded motifs: a closed ring motif in β-ket *versus* infinite chains of hydrogen bonds in the chiral α-ket structure. However, the overall crystal packing of both forms is surprisingly similar, with close-packed layers of antiparallel-oriented benzo­phenone moieties bound by C—H⋯π interactions. Notably, the most important stabilizing term in the total lattice energies in both instances proved to be the dispersion related to these interactions. Both forms of the title compound (α- and β-ket) were additionally characterized by differential scanning calorimetry and thermogravimetric analysis.

## Abstract 2

The crystal and molecular structures of the popular anti-inflammatory drug ketoprophen were examined. The centrosymmetric form of ketoprophen (β-ket) that has been reported so far, was structurally, geometrically and energetically compared with the non-centrosymmetric form (*S*-enantiomer) of ketoprophen (α-ket). The molecules in both crystal structures are in similar arrangements, with antiparallel-oriented benzo­phenone moieties.

## 1. Introduction   

**Figure 1:**
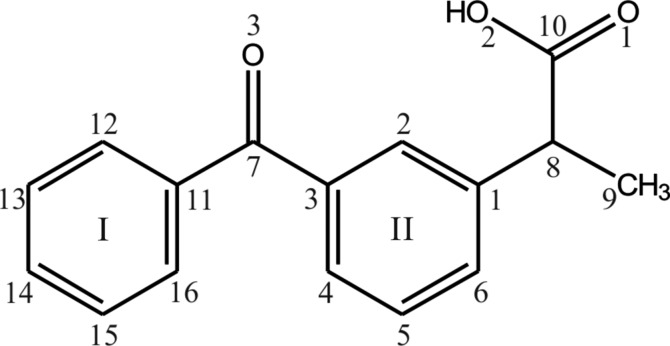
Structure of ketoprophen, (*RS*)-2-(3-benzoyl­phenyl)propionic acid, with numbering of the aromatic ring atoms.

Ketoprophen (Figs. 1 ▸ and 2 ▸) belongs to the family of propionic acid derivatives and because of its specific pharmaceutical properties has found practical application as one of the most commonly used non-steroidal anti-inflammatory drugs (Kantor, 1986 ▸). It is available in many countries, in various forms, under different brand names (Ketonal, Orudis, Ketoflam, Ketomex, Oruvail and others) and is prescribed majorly for treatment of chronic or acute pain (Moore *et al.*, 1998 ▸).

Generally, inhibitors of cyclooxygenase-1 and cyclo­oxygenase-2 enzymes (COX-1 and COX-2) (Hutt & Caldwell, 1984 ▸) such as ketoprophen have a propionic acid core structure. Notably, ketoprophen has two enantiomers which exhibit significantly different biological activity. The (*R*)-enantiomer inhibits cyclo­oxygenase quite weakly, and its analgesic effect depends on bioinversion to the (*S*)-form after ingestion (Aberg *et al.*, 1995 ▸). For the above reason, the pure (*S*)-enantiomer, or the racemic mixture, is preferred for pharmaceutical applications (Beltrán *et al.*, 1998 ▸). Ketoprophen can also be an interesting object of studies involving polymorphism, crystal structure prediction or crystal engineering (Schultheiss & Newman, 2009 ▸). A detailed search of the Cambridge Structural Database (CSD version 5.37) revealed that until now, only the structure of the racemic mixture has been deposited and described (Briard & Rossi, 1990 ▸). The lack of structures of pure enantiomers, especially the (*S*)-enantiomer, is quite surprising, considering its pharmaceutical importance. As in the case of a great number of other pharmaceuticals, existing crystallographic studies are based on the independent atom model (IAM), which assumes sphericity of atoms and neglects quantitative charge-density details (Coppens, 1997 ▸). However, the specific pharmaceutical properties of chemical compounds may also depend on their electronic properties. What is essential, information about details of electron density, is inaccessible in the case of routine crystallographic studies (Espinosa *et al.*, 1999 ▸). In order to obtain improved molecular geometries and quantitative distributions of charge densities in the crystal, high-resolution crystallographic data must be obtained and an experimental charge-density analysis must be performed (Gryl *et al.*, 2011 ▸). Approaches of this type offer a better understanding of the role of molecular interactions in the crystal structure and allow analysis of the electrostatic properties of the compounds which may be linked to their specific biological activity (Schmidtmann *et al.*, 2009 ▸). The results of our studies combine a detailed description of both the racemic mixture and the (*S*)-enantiomer of ketoprophen, a topological charge-density analysis and a detailed description of the molecular interactions occurring in the crystal structure of both forms. The crystal and molecular structure of the (*S*)-enantiomer will be described here for the first time and the interactions that stabilize its crystal structure will be compared with those present in the enantiomeric form. Both forms of the compound were analyzed using differential scanning calorimetry (DSC) and thermogravimetric analysis (TGA).

## 2. Experimental   

### 2.1. Materials   

**Figure 2:**
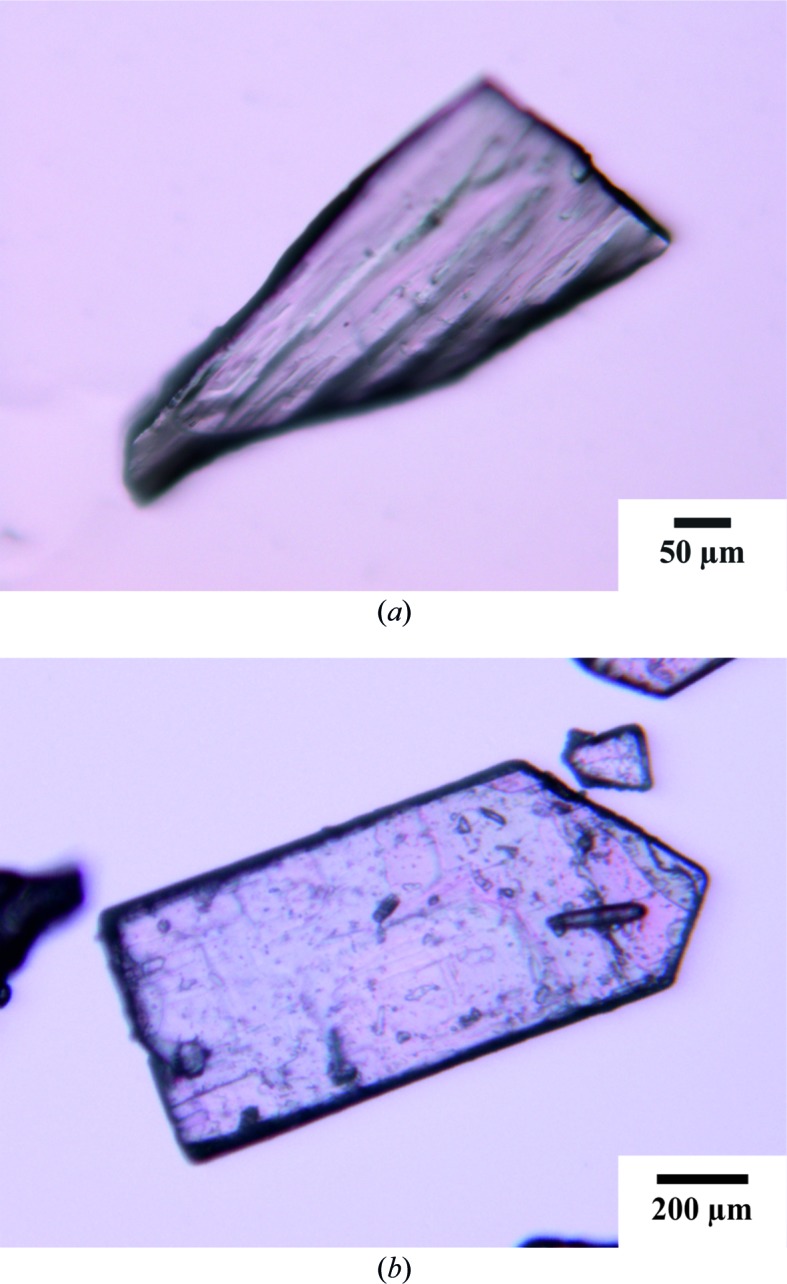
Single crystals of (*a*) β-ket and (*b*) α-ket.

Two single-crystal specimens were examined (Fig. 2 ▸). The (*S*)-ketoprophen was purchased from Sigma–Aldrich (batch No. 471909). An appropriate single crystal was selected directly from the commercial reagent and named α-ket [the (*S*)-enantiomer]. In the case of the racemic form, good-quality single crystals were obtained after recrystallization of the contents of a ‘Ketonal’ capsule (Novartis, β-ket). The structure of (*RS*)-ketoprophen from the Cambridge Structural Database (CSD refcode: KEMRUP) was also compared with our current results. A racemic mixture of the title compound for thermoanalytical research was purchased from Abcam.

### 2.2. Crystal structure determination   

Single-crystal X-ray diffraction data for α-ket and β-ket were collected on a Bruker Apex II CCD diffractometer (Bruker AXS Inc., Madison, WI, USA) using a rotating anode X-ray source (Mo *K*α radiation, λ = 0.71073 Å) equipped with Oxford Cryosystem nitro­gen gas-flow apparatus and mirror optics. The data were collected at 100 K. The frames were integrated with the Bruker *SAINT* (Bruker AXS Inc., Madison, WI, USA) software package using a narrow-frame algorithm and corrected for Lorentz and polarization effects. Absorption effects were corrected using a numerical method for α-ket and a multi-scan method for β-ket. The data for α-ket were merged using *SORTAV* (Blessing, 1995 ▸). The structures were solved using direct methods (Sheldrick, 1990 ▸) and refined with *SHELXL* (Sheldrick, 2008 ▸) within the graphical interface of *OLEX2* (Dolomanov *et al.*, 2009 ▸). The hydrogen atoms were discernable on the Fourier maps and refined using the riding-atom approximation for the C—H and O—H groups. For β-ket, some restraints and constraints were used (*e.g.* FLAT, SADI, RIGU and SIMU) to model slight disorder (occupancy < 0.1) in the propionic moiety. Rigid-body (RIGU) restraints were imposed on the fragment from C1C to C10C and also for the O1C, O2C and O3C atoms, with an uncertainty of 0.001 Å (Thorn *et al.*, 2012 ▸). For the fragment from the C1C atom to the C6C atom, the planarity restraint (FLAT) was used with an uncertainty of 0.1 Å. The distances C3A–C2A and C3C—C2C, C2A—C1A and C2C—C1C, C6A—C1A and C6C—C1C, C6A—C5A and C6C—C5C, C5A—C4A and C5C—C4C, C4A—C3A and C4C—C3C, C8A—C1A and C8C—C1C, C8A—C10A and C8C—C10C, C7A—C3A and C7C—C3C, and O3A—C7A and O3C—C7C were restrained to be similar (SADI) with an uncertainty of 0.002 Å. Anisotropic displacement parameters were also restrained to be similar (SIMU) for atoms from C8A to C10A, and for O1A and O2A. An illustration of the labelling scheme for the molecule with the disordered fragment is attached in the supporting information (Fig. S1).

The structure of α-ket was also refined with a transferable aspherical atom model (TAAM) for electron density, using *XD2016* (Volkov *et al.*, 2016 ▸). Specific atom types from the University of Buffalo Databank (UBDB) (Jarzembska & Dominiak, 2012 ▸) were transferred using *LSDB* (Volkov *et al.*, 2004 ▸). The atomic displacement parameters (ADPs) for hydrogen atoms were estimated using *SHADE3* (Madsen, 2006 ▸) and the *X*—H distances were averaged values taken from neutron diffraction (Allen & Bruno, 2010 ▸). In the TAAM refinement, the scale factor, atomic positions and ADPs for the non-hydrogen atoms, as well as the valence populations for all the atoms were varied. Applied weights were imported from the *SHELX* refinement. The selected structural information is given in Table 1 ▸. Detailed structural information is available in the supplementary CIFs [see also CCDC references: 1824624 (β-ket), 1824625 and 1824626 (α-ket)]. Unrestrained refinement of the aspherical charge-density model was not attempted because of the limited resolution of X-ray data obtained. The resulting charge-density model is characterized by relatively low residual density values (0.30/−0.29 e Å^−3^) and a random distribution of residual density features (Fig. S3).

### 2.3. Thermogravimetric analysis and differential scanning calorimetry   

TGA/DSC measurements were performed using Mettler-Toledo TGA/DSC *STAR^e^* system: the racemic mixture and (*S*)-enantiomer samples weighing 7.15 and 7.74 mg, respectively, were ground using an agate mortar and pestle and placed in standard alumina 70 µl crucibles (covered with alumina lids with pinholes) and heated at a rate of 10°C min^−1^ under a dry N_2_ atmosphere at a constant flow (50 ml min^−1^) over the range 25–700°C. Obtained data were analysed using *STAR^e^* (Mettler, Toledo). As the crystallization process gave a very limited yield of single crystals, TGA/DSC analysis of the racemic form of the title compound was performed using (*RS*)-ketoprophen reagent purchased from Abcam.

### 2.4. Computational calculations   

Periodic geometry calculations were carried out after IAM refinement for β-ket (major and minor molecules) and after TAAM refinement for the geometry of α-ket with *Crystal09* (Dovesi, Orlando *et al.*, 2009 ▸; Dovesi, Saunders *et al.*, 2009 ▸) using DFT with the B3LYP functional and 6-31 d1G (Gatti *et al.*, 1994 ▸) basis set, and the crystal lattice and dimer interaction energies were calculated with Grimme dispersion (Grimme, 2004 ▸, 2006 ▸) and BSSE correction (Boys & Bernardi, 1970 ▸) applied. The crystal lattice and dimer interaction energies were also computed with PIXEL (Gavezzotti, 2003*a* ▸,*b* ▸, 2002 ▸, 2005 ▸) at MP2/6-31G** level of theory for both α-ket and β-ket using the coordinates obtained from geometry optimization from *Crystal09*. The total interaction energy was divided into four components: electrostatic, dispersion, repulsion and polarization. Intermolecular interaction energies and the resulting energy frameworks were also calculated using *CrystalExplorer17* (Turner *et al.*, 2017 ▸) using the B3LYP functional and 6-31 G(d,p) basis set (Turner *et al.*, 2015 ▸).

## 3. Results and discussion   

### 3.1. Structural information   

**Figure 3:**
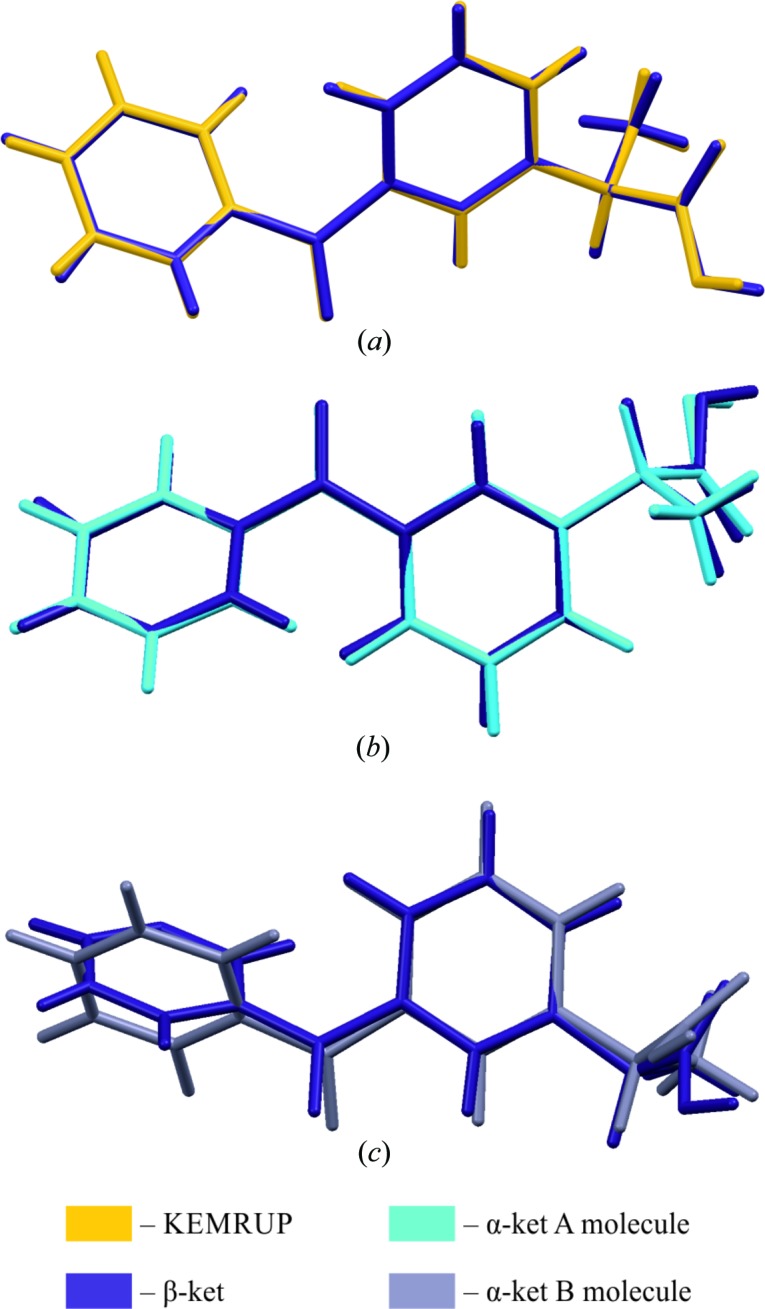
The conformational differences visualized by superpositions of molecules: (*a*) KEMRUP and β-ket, (*b*) β-ket and α-ket molecule A, and (*c*) β-ket and α-ket molecule B. RMSD values [calculated in *Mercury* (version 3.8; Macrae *et al.*, 2006 ▸)] are equal to (*a*) 0.0795, (*b*) 0.7174 and (*c*) 1.1643 Å.

KEMRUP and β-ket crystallized in the 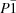 space group with one molecule in the asymmetric unit. For the β-ket molecule, disorder was observed in the propionic acid moiety (Fig. S1). The minor site had only ∼10% occupancy and therefore was refined with numerous restraints, resulting in a less reliable geometry. Hence, we will limit further discussion to the major conformer. The conformational differences between the major β-ket conformer and KEMRUP are illustrated in Fig. 3 ▸(*a*).

The geometrical parameters of the β-ket and KEMRUP molecules are almost identical (RMSD = 0.0795 Å). The deposited KEMRUP structure is missing one hydrogen atom which should be connected to the C15 atom. For further analysis, especially quantitative analysis of intermolecular interactions, the major conformer of our β-ket structure was used.

**Figure 4:**
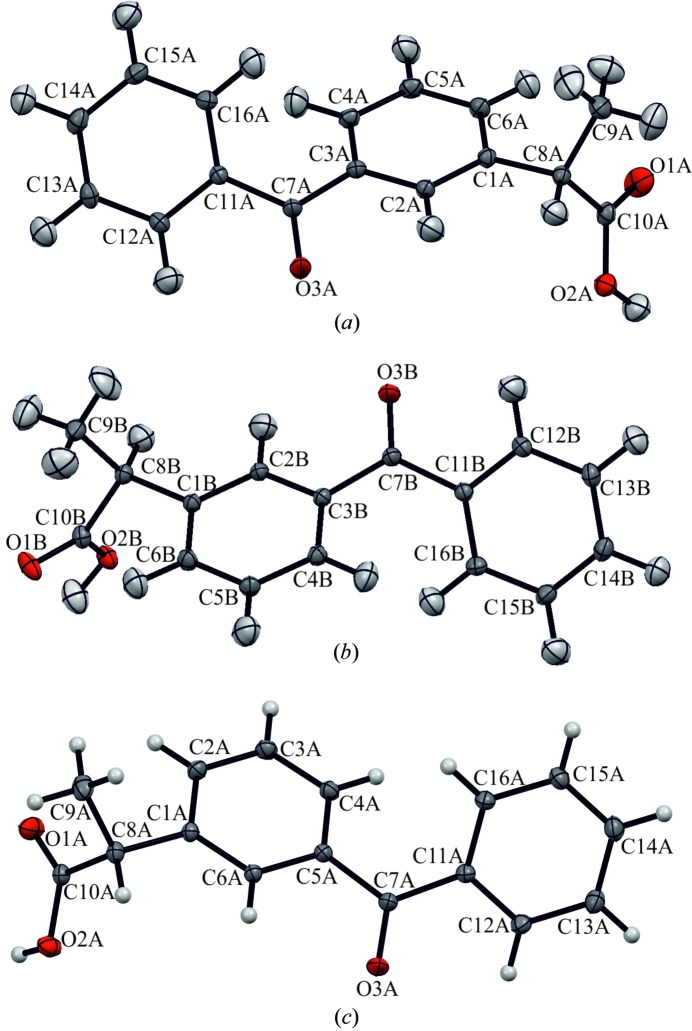
Molecular structures with atom labelling schemes. Atomic displacement parameters are drawn at the 50% probability level for (*a*) the α-ket molecule A, (*b*) the α-ket molecule B and (*c*) the β-ket major conformer. Atomic displacement parameters for H atoms of α-ket were estimated using *SHADE3*.

The α-ket crystallized in the chiral *P*2_1_2_1_2_1_ space group with two molecules in the asymmetric unit. The lengths of the *a* and *b* crystal axes were very similar in both crystal forms, while the length of *c* in α-ket was almost exactly four times the *c* length in β-ket (Table 1 ▸). Taking into account that the cell angles in triclinic β-ket were very close to orthorhombic 90°, certain similarities in the crystal packing of α-ket and β-ket can be expected. The molecular structures with the atom labelling scheme are presented in Fig. 4 ▸.

The conformational differences between β-ket major and the α-ket molecules A and B are shown in Figs. 2 ▸(*b*) and 2 ▸(*c*), respectively. The selected torsion-angle parameters and ring I and II dihedral-angle values for KEMRUP, β-ket and α-ket are summarized in Table 2 ▸.

The ketoprophen two-ring system was not planar in any crystal form and the dihedral angle between the planes containing rings I and II was around 51.5° irrespective of the crystal form (Table 2 ▸). The propionic acid fragment and the methyl group were twisted in opposite directions with respect to ring I in α-ket (Table 2 ▸). The geometry of the α-ket A molecule was much more similar to β-ket than the α-ket B molecule, as illustrated by the respective overlays of molecular structures (Figs. 3 ▸ *b* and 3*c*). The conformational differences between the β-ket minor and the α-ket molecules A and B are shown in Figs. S2(*a*) and S2(*b*) of the supporting information. The bond lengths and angles describing the geometry of α-ket were very similar to those of the already deposited (*RS*)-ketoprophen (Table 3 ▸). All C=O bonds were around ∼1.21 Å, which is a typical value for these bonds. The bond lengths for C—O (∼1.31 Å) were slightly shorter than the typical C—O bond length (∼1.37 Å) (Madsen *et al.*, 1998 ▸). More detailed geometrical information can be found in the supplementary CIFs [see also CSD references: 1824624 (β-ket), 1824625 and 1824626 (α-ket)].

**Figure 5:**
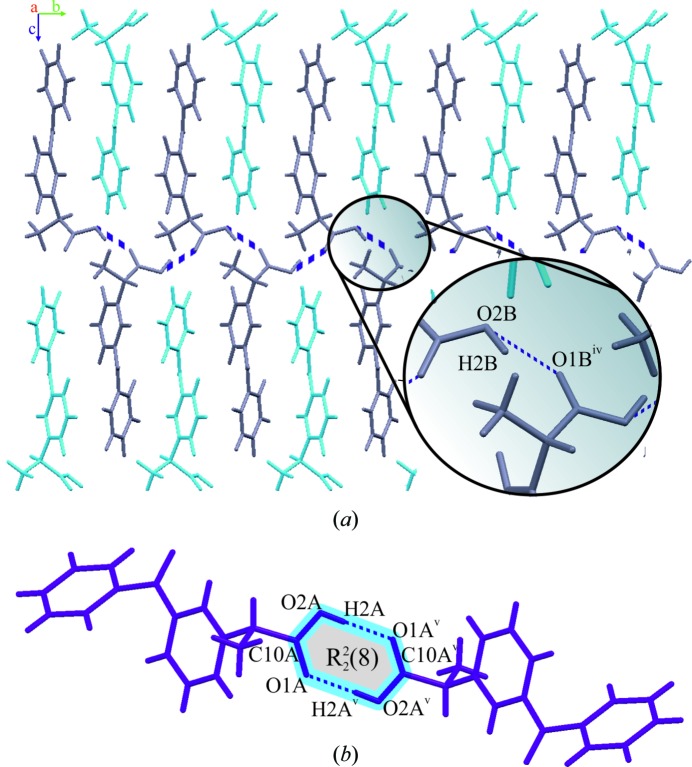
Hydrogen-bonded motifs for (*a*) α-ket and (*b*) β-ket. Symmetry codes: (iv) −*x* + 1, *y* − 1/2, *z* − 3/2; (v) −*x* + 2, −*y* + 1, −*z*.

**Figure 6:**
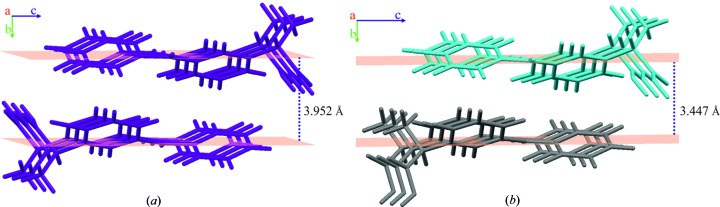
Crystal structure stabilizing interactions in (*a*) β-ket and (*b*) α-ket marked as a light-blue line between the layers. The distance was measured between the average planes of the subsequent molecular layers perpendicular to the [010] direction.

In the β-ket crystal structure, the molecules involved in the O—H⋯O hydrogen bonds were arranged in a ring motif 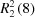 (Etter *et al.*, 1990 ▸) (Fig. 5 ▸) and created a C(5) chain (Etter *et al.*, 1990 ▸) of C=O⋯H interactions in the [100] direction with an O⋯O distance of 2.6236 (6) Å and an O⋯H distance of 2.6606 Å. This crystal structure can be divided into layers in the [100] crystallographic direction with a very interesting arrangement; each consecutive layer has alternately directed molecules, related by the centre of inversion −*x* + 1, −*y* + 1, −*z* + 1 (Fig. 6 ▸). Interlayer interactions were dominated by C—H⋯π contacts with a distance of 3.952 Å.

The crystal packing of α-ket was controlled by O—H⋯O intermolecular interactions and was stabilized by C—H⋯O and C—H⋯π close contacts. Molecules A and B formed alternately arranged comb-like layers in the crystal structure (Fig. 5 ▸), creating C(4) chains (Etter *et al.*, 1990 ▸) of hydrogen bonds along the [100] and [010] crystallographic directions, respectively, with O⋯O distances of 2.698 (2) and 2.659 (2) Å. There were also C—O⋯H contacts observed between A molecules and between B molecules, with O⋯H distances of 2.6468 and 2.6093 Å, respectively. It is worth noting that the global arrangement was very similar to the β-ket crystal structure; there were also layers with C—H⋯π contacts between them, the shortest interlayer distance being 3.447 Å (Fig. 6 ▸). However, the molecules were not related by a centre of inversion, instead the A and B molecules appear in an alternating pattern. Additional short contacts between the propionic acid moiety and aromatic ring I also occurred.

### 3.2. Hirshfeld surface analysis   

Hirshfeld surfaces of molecules (Spackman & Jayatilaka, 2009 ▸) in the crystal were obtained by partitioning space into regions such that a contribution from a given region to the electron density of the crystal derived from the sum of the spherical atoms of a given molecule (promolecule) prevails over the contributions from electron density of any other molecule in the crystal (procrystal). A point in space belongs to the Hirshfeld surface when the promolecule contribution of electron density at this point is equal to the corresponding total electron density of the procrystal. This encloses a region around the molecule where the molecular weight function *w*(**r**) ≥ 0.5,
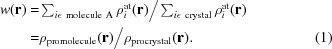
Mapping of interatomic contacts on the Hirshfeld surface was possible with a pair of parameters, *d*_i_ and *d*_e_. The mentioned parameters were the closest distances from the Hirshfeld surface to the atom nucleus located inside the surface *d*_i_ (internal), or outside the surface *d*_e_ (external) (McKinnon *et al.*, 2004 ▸).

**Figure 7:**
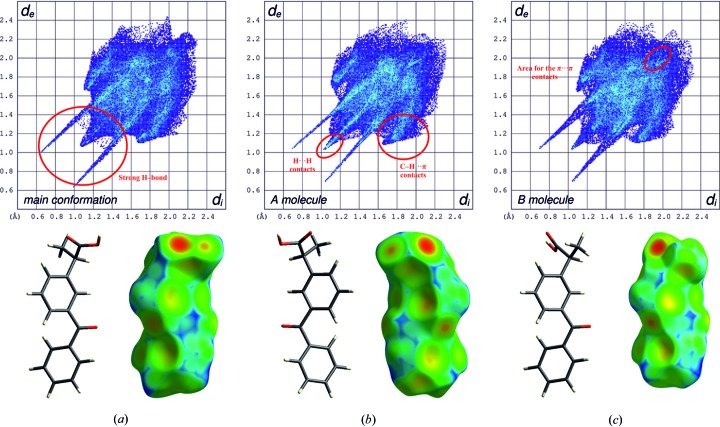
Hirshfeld surfaces mapped onto *d*_e_ and two-dimensional fingerprint plots for the ketoprophen crystal structures of α-ket and β-ket (intermolecular contacts are closer than the sum of their van der Waals radii are highlighted in red on the *d*_e_ surface, longer contacts are blue and contacts around this sum are green).

**Figure 8:**
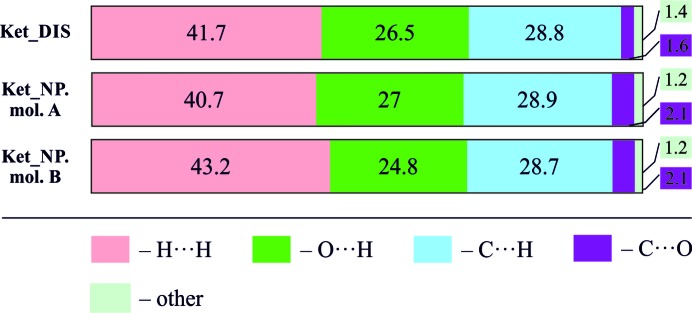
Percentage contributions to the Hirshfeld surface area of the intermolecular contacts for α-ket and β-ket.

The qualitative and quantitative intermolecular contacts that occur in the crystal can be visualized using a two-dimensional fingerprints plot (Spackman & McKinnon, 2002 ▸). These fingerprint plots can help to quickly pinpoint subtle differences in crystal packing between compared crystal structures. Hirshfeld surfaces and two-dimensional fingerprint plots for the β-ket major conformer and for two molecules of α-ket are shown in Fig. 7 ▸ (detailed information is present in Figs. S5–S12). The crystal structures of both forms of ketoprophen were dominated by weak van der Waals interactions. Two-dimensional fingerprint plots illustrate in a concise way the intermolecular interactions in the crystal structure. For all molecules, the C—H⋯π contacts were not clearly associated with the centroid of the benzene ring and this is illustrated as yellow spots around the rings on the *d*_e_ surface, slightly shifted to the left or to the right relative to the centre of the ring. These interactions are observable on the fingerprint plots as characteristic ‘wings’. The positions of the wings clearly indicate that the C—H⋯π contacts were slightly longer in the β-ket major conformer (*d*_C⋯H_ ∼2.8 Å) than in α-ket for both the A and B molecules (*d*_C⋯H_ ∼2.73 Å). The short H⋯H interactions are represented by a small spike on the two-dimensional fingerprint plot around 1.03 or 1.1 Å, where *d*_e_ is almost equal to *d*_i_ for A or B molecules and the major conformer of β-ket, respectively. For these crystalline forms, the π⋯π interactions in the crystal were absent. A characteristic of the π⋯π interactions in the two-dimensional fingerprint plots was a feature around *d*_e_ = *d*_i_ ≃ 1.7–1.8 Å and for both forms this feature was shifted to almost 2 Å; this is denoted in red in Fig. 7 ▸(*c*). The shapes of the Hirshfeld surfaces and fingerprint plots for the crystal structures were very similar. In both cases, each molecule participated in two equivalent hydrogen bonds. The Hirshfeld surface area mapped onto *d*_e_ for the hydrogen bonds was located near the carb­oxy­lic acid groups. A large red spot appears for the hydrogen acceptors and a small yellow or orange-red spot is present for the donor (McKinnon *et al.*, 2004 ▸) in Fig. 7 ▸. For both forms, this region on the Hirshfeld surface differs considerably. For the major conformer of the β-ket molecule, this area characteristically reflects the cyclic hydrogen bonds – the dots for the acceptors and donors of the hydrogen atom are adjacent. This feature was not observed for the A and B molecules of α-ket. The two-dimensional fingerprint plots (Fig. 7 ▸) contain the expected hydrogen-bonding sharp spikes, and for the major conformer of β-ket, a diffuse area of points between these spikes was present with a shortest contact near 2.4 Å (Fig. 7 ▸ *a*). These points correspond to the very close H⋯H contacts across the hydrogen-bonded motif. The fingerprint plot of the major conformer of β-ket showed an unambiguously shorter hydrogen bond (*d*_H⋯O_ ∼1.62 Å) than in the A and B molecules of α-ket (*d*_H⋯O_ ∼1.72 Å). The spikes in molecule B were broader than in molecule A. The summation of the percentage contribution to the Hirshfeld surface area for intermolecular contacts is present in Fig. 8 ▸. These numbers illustrate a great similarity of the studied structures. The biggest percentage contributions to the Hirshfeld surface were the H⋯H, O⋯H and C⋯H contacts.

### 3.3. Topological charge-density analysis   

**Figure 9:**
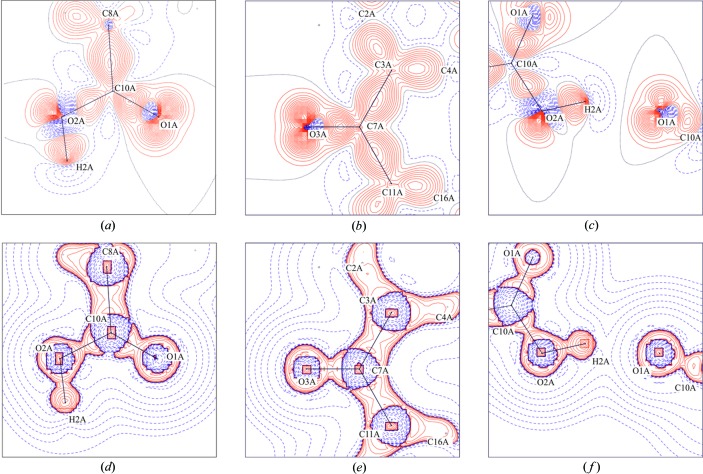
Deformation density maps [(*a*), (*c*), (*e*) contour ±0.05 e A^−3^] and their negative Laplacian [(*b*), (*d*), (*f*) contour values in geometric order, starting value ±0.1, increments of 2] for the experimental multipolar model. Red (solid lines) and blue (broken lines) colours represent positive and negative contours, respectively.

The critical points for bonds, rings and hydrogen bonds in α-ket were searched using *XD2016* and are shown in Fig. S13, and selected values are listed in Tables 4 and 5 (a full list of bond critical point (BCP) values is given in Table S1). For the benzene ring, the ρ_BCP_ for C—C bonds ranged from 2.072 to 2.110 e Å^−3^ and the 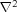
ρ_BCP_ from −17.188 to −18.146 e Å^−5^. These are similar to literature values obtained both experimentally (Jeffrey & Piniella, 2012 ▸) and theoretically (Bader, 1994 ▸). The ring critical points were located in the centres of all the rings. The critical-point properties for the C—H bonds from both methyl groups were very similar to the C—H bonds from the benzene ring. The small ellipticity (around 0.01–0.02) of these bonds highlights their single-bond nature. From a chemical point of view, the C3A—C7A, C7A—C11A, C3B—C7B and C7B—C11B bonds should be single. The values of ρ_BCP_ and ellipticity for all these bonds are smaller than for C—C in the benzene ring. However, ε is larger than for formal single bonds (ε = 0.09 in all cases), which is expected for atoms linked with an unsaturated system (Bader *et al.*, 1983 ▸). For the C7A—O3A and C7B—O3B bonds, the ρ_BCP_ had a value around 2.823 e Å^−3^ and 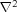 ρ_BCP_ ranges from −23.573 to −24.026 e Å^−5^. However, the ellipticity of these bonds (*ε* = 0.02) was lower than expected for double bonds. The examination of the interactions in the crystal structure suggested a transfer of electrons between the benzene rings and the C=O group. For the C1A—C8A, C8A—C9A, C8A—C10A, C1B—C8B, C8B—C9B and C8B—C10B bonds, ρ_BCP_ was in the range from 1.590 to 1.767 e Å^−3^ and the 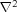
ρ_BCP_ from −10.722 to −13.813 e Å^−5^. The ellipticities for these bonds were equal to zero, except for the C8A—C10A and C8B—C10B bonds (where *ε* = 0.08, which may be related to the proximity of the hydrogen bonds). The critical points (CPs) in the C—O and O—H bonds were located closer to the carbon or hydrogen atoms than to oxygen, which is more electronegative. The deformation density maps and Laplacian maps for the carboxylic moieties, carbonyl moieties and the hydrogen-bonded system are presented in Fig. 9 ▸.

The lone-pair electrons of the oxygen atoms and bonding densities of the C—C and C—O bonds are clearly visible. The Laplacian values at CPs for C—O had a large negative value of the Laplacian (Table 4 ▸) and are shown in Fig. 9 ▸(*d*) as a large red area between the carbon–oxygen atoms with characteristic narrowing. For both molecules, ρ_BCP_ values for the hydrogen bonds were small and conventional (Carroll & Bader, 1988 ▸) (∼0.257 e Å^−3^). The 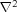
ρ_BCP_ values were small and positive (∼2.702 e Å^−5^), identifying all of them as closed-shell interactions. The total energy density *H*_r_ for all hydrogen-bond critical points took a small, negative value which corresponds to medium-strength interactions according to the work by Rozas *et. al*. (2000 ▸).

The source function of the electron density was proposed by Bader & Gatti (1998 ▸) as defining the contribution from each atom to the electron density at a specific point (*e.g.* at a hydrogen-bond critical point). In this regard, the integration over the local source of electron density in the atomic basin generates the electron density ρ(**r**) as a sum of the atomic contributions. The source function is very sensitive to any perturbations and can show subtle local electron density changes. It is therefore very useful as a descriptor for characterization of bonding features (Farrugia *et al.*, 2006 ▸), especially hydrogen bonds.

The percentage contributions from the source function to ρ_BCP_ for selected hydrogen bonds in α-ket are summarized in Table 5 ▸. In all cases, the source contributions from the donors slightly exceed that from the acceptors. The source contributions from hydrogen atoms were small and negative (around −9%). The source contributions from H and *A* atoms *S*(*A* + H) were positive and smaller than 50%, which indicated that the hydrogen-bond critical points act more like a sink than a source of electron density (Gatti *et al.*, 2003 ▸). The source contributions from the triad (*D* + H + *A*) suggest that these hydrogen bonds were medium-strength, which is in a good agreement with the Rozas rules (Rozas *et al.*, 2000 ▸). The percentage of source contribution from H, *D*, *A*, (*A* + H), (*D* + H) and (*D* + H + *A*) atoms were characteristic for hydrogen bonds with polarization assistance (Gatti *et al.*, 2003 ▸).

### 3.4. Electrostatic potential and net atomic charges   

**Figure 10:**
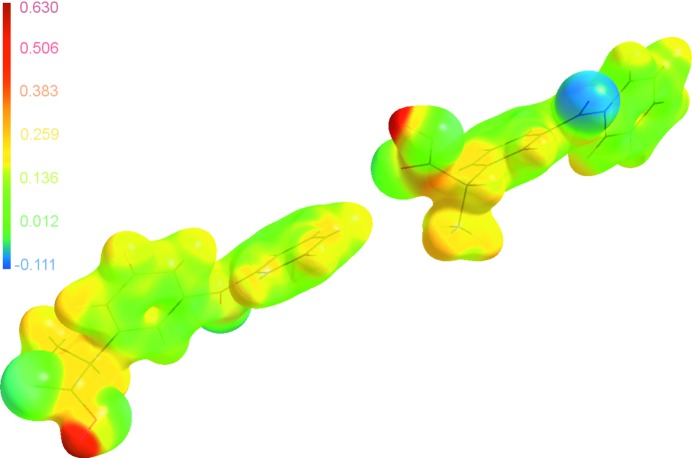
Electrostatic potential (e A^−1^) mapped onto the 0.1 e A^−3^ electron-density isosurface. The potential at +0.630 e A^−1^ is shown in dark red and −0.111 e A^−1^ is shown in blue.

The electrostatic potential mapped onto the electron-density isosurface around the molecules was calculated according to the Su and Coppens method (Su & Coppens, 1992 ▸) based on the populations of the multipoles found in the multipolar refinement. In Fig. 10 ▸, a map of electrostatic potential is shown for both α-ket molecules mapped with *MolIso* (Hübschle & Luger, 2006 ▸). The spaces coloured red, blue and green represent electropositive, electronegative and neutral regions, respectively. The carbonyl oxygen from the keto group marks the most electronegative area in both molecules. However, strongly electronegative regions were also present near the oxygen atoms of the carboxyl group. They are indicated as pale blue–green regions on the maps. Positive polarization on carboxyl, methyl and phenyl hydrogen atoms are marked as red, pale orange and yellow regions, respectively.

The net atomic charges obtained by several different methods are summarized in Table S2. Not surprisingly, according to each method, the oxygen atoms retained very similar negative charges.

### 3.5. Interaction energies   

**Figure 11:**
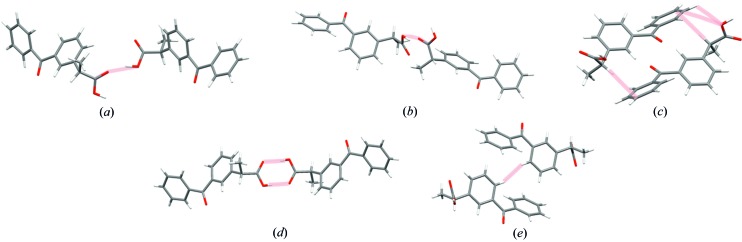
Dimers of interacting molecules (*a*) A⋯A, (*b*) B⋯B and (*c*) A⋯B for α-ket, and (*d*) A⋯A and (*e*) C–H⋯π for β-ket.

Many interesting interatomic interactions could be identified in the crystal structure of the investigated compounds. The molecules involved in these interactions are shown in Fig. 11 ▸ for α-ket (*a*)–(*c*) and β-ket (*d*)–(*e*). The (*S*)-enantiomer crystallized with two molecules in the asymmetric unit (A and B molecules), so the A⋯A and B⋯B dimers, involved in the O—H⋯O contacts, and also the A⋯B dimer with C—H⋯π interactions were examined. On the other hand, (*RS*)-ketoprophen crystallized with one molecule in the asymmetric unit and the A⋯A dimer resulted from O—H⋯O interactions. The dimer interaction energies were computed with *Pixel*, *Crystal* and *CrystalExplorer* for α-ket and β-ket (major and minor conformations), and are summarized in Table 6 ▸ (information about dimer energies for the minor disorder component is attached in Table S3).

The total lattice energy was calculated using *Pixel* and *Crystal* approaches for both α-ket and β-ket. Generally, these methods showed good agreement. Both methods assigned slightly lower lattice energies to β-ket, suggesting a more favourable energy of crystallization compared with that of the pure (*S*)-enantiomer. The differences in lattice energies were, however, very small: about −4 kJ mol^−1^ (computed with *Pixel*) and about −8 kJ mol^−1^ (computed by *Crystal*) (Table 7 ▸; for β-ket minor conformer see in Table S3). Notably, the most important stabilizing term in the total lattice energy was dispersion, related to C—H⋯π interactions, irrespective of the crystal form of ketoprophen. Electrostatic terms that are related to the formation of O—H⋯O interactions played a secondary role.

According to the performed calculations, the total dimer interaction energy in α-ket was lower for B⋯B than A⋯A by about −10, −8 or −6 kJ mol^−1^ (results from *Crystal*, *Pixel* or *CrystalExplorer*, respectively), which is comparable with the energies of C—H⋯π-stabilized A⋯B dimers. The results from *Crystal* suggested that the A⋯B interaction energy was close to that of A⋯A, whereas *Pixel* yielded energies closer to the B⋯B dimer. According to *CrystalExplorer*, the A⋯B dimer had the lowest dimer interaction energy.

The electrostatic energies for the B⋯B dimer in α-ket were larger than for the A⋯A dimer, implying slightly stronger hydrogen bonding. The differences between these energies were −10 and −5 kJ mol^−1^, obtained from *Pixel* and *CrystalExplorer*, respectively. For both the A⋯A and the B⋯B dimer interactions, the electrostatic term was the most stabilizing. In the case of repulsion interactions, the larger contribution to the total dimer energy was also that of the B⋯B dimer, probably because of steric hindrance between the carb­oxy­lic groups of the B⋯B contacts in the [010] direction.

For the A⋯B interactions, the electrostatic term was secondary to the dispersive term. The latter reached quite large values, similar to the electrostatic terms in the A⋯A or B⋯B dimers (about −58 or −62 kJ mol^−1^ calculated by *Pixel* or *CrystalExplorer*, respectively).

In contrast, for the β-ket compound, the values of the total interaction energies for the A⋯A dimer were two to three times higher than for the A⋯A dimer in the α-ket crystal structure, depending on the computational approach. This is not very surprising considering the ring-forming, double hydrogen-bonded motif connecting the β-ket molecules. The A⋯A or B⋯B dimers in α-ket are stabilized by a single hydrogen bond. In particular, the electrostatic interaction term for the A⋯A dimer in β-ket was more than three times (*Pixel* and *CrystalExplorer*) larger for the A⋯A dimer in the α-ket crystal structure.

On the other hand, the total energy of the C—H⋯π interaction was almost the same in β-ket and the A⋯B dimer in α-ket, irrespective of the computational approach, making it of the same magnitude as the total interaction energy of the A⋯A dimer, analogous to the α-ket case. The similarity was still preserved when the electrostatic, dispersion and repulsion terms were analysed separately with *Pixel* or *CrystalExplorer*. The contribution from the dispersion term had the dominant stabilizing effect of about −56 or −63 kJ mol^−1^ according to *Pixel* and *CrystalExplorer*, respectively; these values are almost identical to those registered for the A⋯B dimer in α-ket. The electrostatic term for the C—H⋯π interactions in β-ket also had very consistent values independent of the computational approach. The uniformity of the C—H⋯π interactions between β-ket and α-ket reflected the fact that very similar, layer-based packing arrangements were adopted by both crystal forms. This was also observed for the minor conformer within the β-ket crystal packing (as documented in Table S3 and Fig. S14).

**Figure 12:**
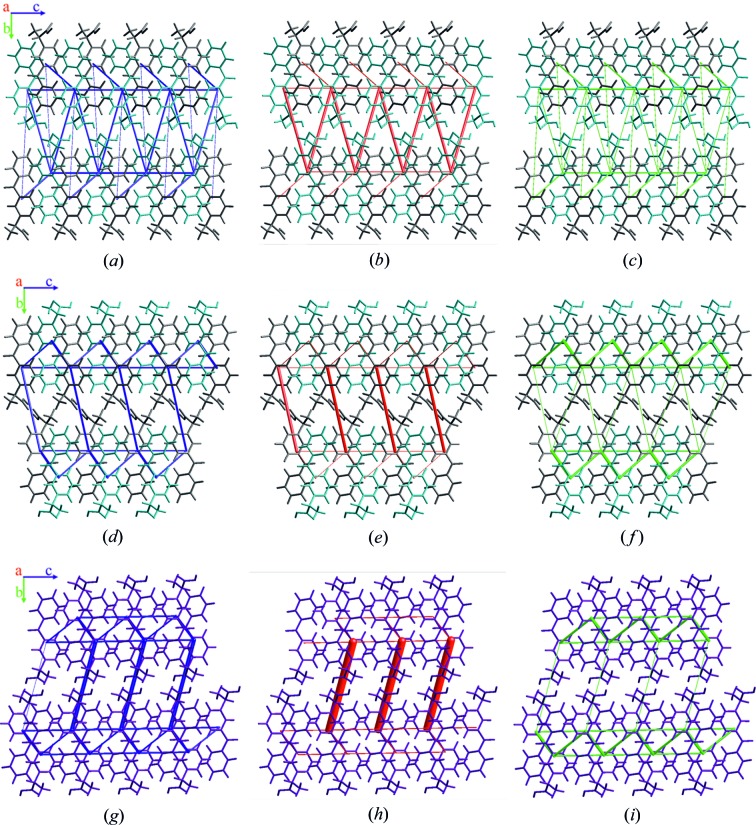
Energy frameworks for the crystal structure of the (*S*)-enantiomer: (*a*)–(*c*) A molecule, (*d*)–(*f*) B molecule and (*g*)–(*i*) the racemic mixture (major conformer). The tube size is 36 arbitrary units and the cut-off is 8 kJ mol^−1^. Colouring scheme: total energy = blue, electrostatic energy = red and dispersion energy = green.

The visualization of intermolecular interactions described above and their electrostatic and dispersion components for both crystal forms are shown in Fig. 12 ▸ as energy frameworks calculated by *CrystalExplorer17* (an analogous visualization for the β-ket minor conformer is presented in Fig. S14). Energy frameworks confirmed that the electrostatic contribution to the total dimer interaction energy was higher for dimers involved in hydrogen bonding, whereas the dispersion contribution was dominant for the C—H⋯π interactions. It is worth noting that the overall energy framework pattern for the α-ket molecule B was more similar to β-ket than the α-ket molecule A, even though molecule A showed greater similarity to β-ket on a molecular level. However, even for the A molecule of α-ket, the general pattern in the energy framework was very similar to β-ket, although the interactions were slightly less directional and more symmetric. Apparently in ketoprophen, there exists a preferred crystal-packing architecture that more closely resembles the β-ket racemic crystal. The pure (*S*)-enantiomer, devoid of its racemic counterpart, seemed to form a similar crystal architecture by adopting two distinct conformations in the crystal packing.

### 3.6. Thermogravimetric analysis and differential scanning calorimetry   

**Figure 13:**
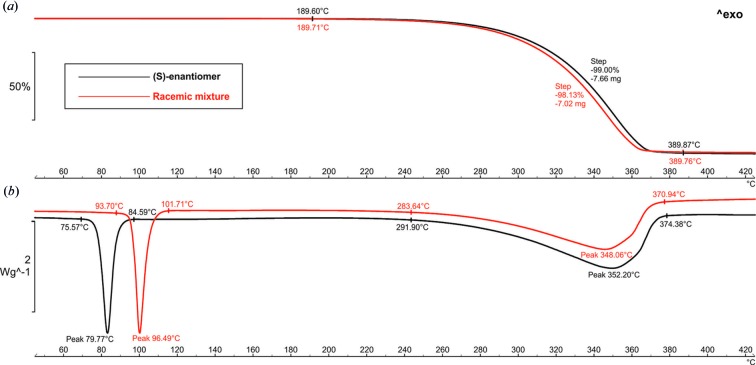
(*a*) TG and (*b*) DSC plots for the racemic mixture and the (S)-enantiomer of ketoprophen.

The DSC curves (Fig. 13 ▸ *b*) showed two endothermic peaks in the case of both forms of the title compound. The endothermic reaction peak, which was not accompanied by weight loss, occurs at 79.77 and 96.49°C for the (*RS*)- and (*S*)-forms, respectively. This reaction was attributed to the melting of the compound. The higher melting point of (*RS*)-ketoprophen may indicate that this form has the largest total crystal lattice energy, which is in agreement with the results of performed theoretical calculations. The broad endothermic peak with its maximum at 348.06 and 352.20°C was attributed to a one step decomposition that was confirmed by the 98.13 and 99.00% weight loss of the sample viewed on the TG curve for the racemic mixture and pure (*S*)-enantiomer of title compound, respectively (Fig. 13 ▸ *a*).

## 4. Summary   

The crystal structure of the pure (*S*)-enantiomer of the popular analgesic and anti-inflammatory drug ketoprophen (α-ket) has been determined. A detailed aspherical charge-density model based on the high-resolution X-ray diffraction data has been refined. Special attention has been paid to the charge-density distribution around the carbonyl or carboxyl groups. The C—H⋯π and O—H⋯O were the most important interactions in the crystal structure of (*S*)-ketoprophen. Detailed examination of the charge-density distributions and bond lengths in these regions confirmed an electron transfer through these contacts. In particular, a careful examination of the topological properties of the electron charge density in the regions of O—H⋯O hydrogen bonds revealed their medium-strength character with polarization assistance.

In order to compare the crystal structure of the (*S*)-enantiomer of ketoprophen with the racemic mixture, the structure of the latter form has also been re-determined at 100 K, based on high-resolution data (β-ket). The final structure of the racemate turned out to contain approximately 10% disorder, unreported in previous work. Comparisons of structural features and energetics were carried out between the α-ket and the major, well defined conformer of β-ket.

In contrast to the racemic β-ket case, the (*S*)-enantiomer crystallized with two independent molecules in the asymmetric unit, showed distinct conformations of the propionic moiety and, to a lesser extent, the terminal phenyl moiety. The major resulting differences between the β-ket and α-ket crystal forms were observed as distinct hydrogen-bonding motifs: β-ket presents a two-hydrogen-bonded ring motif characteristic for carb­oxy­lic acids, while infinite chains of hydrogen bonds stretching in separate directions are formed by the two independent molecules of ketoprophen in the non-centrosymmetric α-ket structure. Despite these differences, the overall crystal packing of both forms was very similar, with close-packed layers of antiparallel-oriented benzo­phenone moieties, interacting through C—H⋯π interactions.

Inspection of the Hirshfeld surfaces for both α-ket and β-ket showed that the crystal structures of both forms relied substantially on weak intermolecular interactions such as C—H⋯π, and despite different hydrogen-bonding motifs, the crystal packing of both forms is very similar.

Theoretical calculations proved that α-ket and β-ket have very similar lattice energies. The most important stabilizing term in the total lattice energies in both instances proved to be dispersion related to the C—H⋯π interactions. The electrostatic term, related to the formation of O—H⋯O interactions, plays a secondary role in the stabilization of the crystal structure. A more detailed analysis of the intermolecular interaction energies confirmed that the C—H⋯π interactions stabilize layers of antiparallel-oriented benzophenone moieties in both crystal forms.

Apparently, in the case of ketoprophen, there seemed to exist a preferred crystal architecture that more closely resembled the β-ket racemic crystals which are highly dependent on the close packing of the benzo­phenone moieties. The pure (*S*)-enantiomer, devoid of its racemic counterpart, seemed to form a similar crystal architecture by adopting two distinct conformations in the crystal. The preferred crystal-packing motif was further confirmed by conduction an extensive set of crystallization experiments.

## Supplementary Material

Crystal structure: contains datablock(s) 2015_12_30_am_ket_0m, 2016_03_20_am_ketsigma_0m, xd_refine. DOI: 10.1107/S2052252518013222/lq5013sup1.cif

Supporting figures and tables. DOI: 10.1107/S2052252518013222/lq5013sup2.pdf

CCDC references: 1870885, 1870886, 1870887, 1870888, 1870889, 1870890

## Figures and Tables

**Table 1:** Crystallographic information for the analysed compounds

Compound	KEMRUP	β-Ket	α-Ket
Crystal system	Triclinic	Triclinic	Orthorhombic
Formula	C_16_H_14_O_3_	C_16_H_14_O_3_	C_16_H_14_O_3_
*T* (K)	RT	100	100
Space group	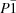	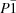	*P*2_1_2_1_2_1_
*a* (Å)	6.136 (2)	6.0671 (4)	6.1130 (5)
*b* (Å)	7.741 (3)	7.5611 (5)	7.3809 (6)
*c* (Å)	13.893 (8)	13.8523 (9)	55.524 (5)
α (°)	88.78 (4)	92.078 (3)	90
β (°)	94.56 (4)	93.838 (2)	90
γ (°)	89.61 (3)	90.994 (3)	90
*V* (Å^3^)	657.639	633.493	2505.2 (4)
*Z*	2	2	8
*R* factor (%)	6.61	3.73	6.12
Resolution (Å)	–	0.5	0.5
ρ_calc_(g cm^−3^)	1.28	1.333	1.348
μ (mm^−1^)	0.726	0.092	0.093
*F*(000)	268	268.0	1072.0
Wavelength (Å)	1.5418	0.71073	0.71073
*R*_int_ (%)		2.91	3.89
			
*SHELX* refinement			
*No.* of parameters	–	282	347
*R*(*F*) all	–	0.0487	0.1113
*wR*2	0.067	0.1202	0.1604
GoF	–	1.044	1.073
Highest peak/hole (e Å^−3^)	0.28/−0.29	0.67/-0.35	0.61/−0.39
			
TAAM refinement			
*R*(*F*)	–	–	0.0382
*R*(*F*2)	–	–	0.0471
*wR*2	–	–	0.0984
GoF	–	–	1.4443
R.m.s. β-ket *vs* α-ket (e Å^−3^)	–	–	0.059
Highest peak/hole (e Å^−3^)	–	–	0.30/−0.29

**Table 2:** Selected torsion-angle parameters (°) and the relative dihedral angles (°) of rings I and II Dihedral angles were calculated using *Mercury* (3.8; Macrae *et al.*, 2006 ▸).

Compound	C10—C8—C1—C2	C9—C8—C1—C6	C6—C1—C3—C16	Ring I and II dihedral
KEMRUP	–116.1 (5)	–55.6 (6)	67.0 (5)	52.7
β-Ket	–117.3 (4)	–57.1 (5)	65.7 (5)	51.9
α-Ket mol. A	–121.0 (1)	–65.5 (2)	–66.5 (2)	50.4
α-Ket mol. B	130.6 (1)	70.0 (2)	66.2 (2)	51.0

**Table 3:** Selected bond lengths (Å) for both crystal forms of investigated compound

Ket (CSD)		α-Ket (mol. A)		α-Ket (mol. B)		β-Ket (major conformer)		β-Ket (minor conformer)	
O1—C10	1.248 (6)	O1—C10A	1.211 (2)	O1B—C10B	1.213 (2)	O1A—C10A	1.2304 (6)	O2C—C10C	1.24 (2)
O2—C10	1.254 (6)	O2A—C10A	1.316 (2)	O2B—C10B	1.318 (1)	O2A—C10A	1.3061 (6)	O1C—C10C	1.31 (2)
O3—C7	1.218 (5)	O3A—C7A	1.214 (2)	O3B—C7B	1.216 (2)	O3A—C7A	1.2245 (6)	O3C—C7C	1.225 (2)
O2—H6	1.024	O2A—H2A	1.012 (1)	O2B—H2B	1.011 (1)	O2A—H2A	0.90 (1)	O2C—H2C	0.840
C1—C6	1.385 (7)	C1A—C6A	1.400 (2)	C1B—C6B	1.400 (1)	C1A—C6A	1.4015 (6)	C1C—C6C	1.39 (1)
C1—C8	1.532 (6)	C1A—C8A	1.523 (2)	C1B—C8B	1.525 (2)	C1A—C8A	1.5222 (6)	C1C—C8C	1.523 (2)
C8—C9	1.519 (9)	C8A—C9A	1.529 (2)	C8B—C9B	1.528 (2)	C8A—C9A	1.5311 (7)	C8C—C9C	1.50 (2)
C8—C10	1.516 (7)	C8A—C10A	1.514 (2)	C8B—C10B	1.514 (2)	C8A—C10A	1.5103 (6)	C8C—C10C	1.511 (2)

**Table 4:** Selected CP topological properties

Bond	ρ (e A^−3^)	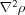 (e A^−5^)	λ1 (e A^−5^)	λ2 (e A^−5^)	λ3 (e A^−5^)	ε	*G*_r_ (Ha a_0_^−3^)	*V*_r_ (Ha a_0_^−3^)	*H*_r_ [Ha a_0_^−3^]	*G*_r_/ρ (Ha e^−1^)	*H*_r_/ρ (Ha e^−1^)	|*V*_r_|/*G*_r_
O3A—C7A	2.826	−23.573	−24.61	−24.15	25.19	0.02	0.510	−1.264	−0.754	1.217	−1.801	2.480
C3A—C7A	1.819	−14.049	−12.89	−11.80	10.64	0.09	0.226	−0.597	−0.371	0.837	−1.378	2.646
C1A—C8A	1.636	−10.814	−11.11	−10.48	10.77	0.06	0.196	−0.504	−0.308	0.807	−1.270	2.573
C5A—C6A	2.092	−17.746	−15.45	−12.88	10.59	0.20	0.285	−0.753	−0.469	0.918	−1.512	2.647
C6A—H6A	1.917	−20.945	−17.36	−16.97	13.38	0.02	0.207	–0.632	−0.425	0.730	−1.495	3.048
C7A—C11A	1.821	—14.107	—12.89	—11.84	10.63	0.09	0.226	−0.598	−0.372	0.837	−1.379	2.648
C8A—C10A	1.767	−13.787	−12.53	−11.62	10.36	0.08	0.212	−0.567	−0.355	0.810	−1.356	2.674
C8A—C9A	1.590	−10.749	−10.73	−10.71	10.68	0.00	0.184	−0.479	−0.295	0.779	−1.252	2.608
C9A—H9AA	1.906	−21.09	−17.04	−16.93	12.89	0.01	0.203	−0.625	−0.422	0.719	−1.493	3.078
O3B—C7B	2.820	−24.026	−24.50	−24.03	24.51	0.02	0.504	−1.257	−0.753	1.206	−1.803	2.495
C3B—C7B	1.811	−13.906	−12.83	−11.73	10.65	0.09	0.224	−0.593	−0.368	0.835	−1.373	2.643
C1B—C8B	1.631	−10.722	−11.03	−10.47	10.78	0.05	0.195	−0.501	−0.306	0.806	−1.267	2.571
C5B—C6B	2.087	−17.628	−15.4	−12.84	10.62	0.20	0.284	−0.751	−0.467	0.918	−1.509	2.644
C6B—H6B	1.923	−21.087	−17.42	−17.03	13.37	0.02	0.208	−0.635	−0.427	0.731	−1.498	3.051
C7B—CC11B	1.815	−13.992	−12.84	−11.8	10.64	0.09	0.225	−0.595	−0.370	0.836	−1.375	2.646
C8B—C10B	1.766	−13.813	−12.55	−11.62	10.36	0.08	0.212	−0.567	−0.355	0.809	−1.356	2.677
C8B—C9B	1.592	−10.787	−10.74	−10.73	10.68	0.00	0.184	−0.480	−0.296	0.779	−1.254	2.609
C9B—H9BA	1.897	−20.817	−16.92	−16.81	12.91	0.01	0.202	−0.620	−0.418	0.719	−1.487	3.068

**Table:** Symmetry codes: (i) *x* − 1/2, −*y* + 3/2, −*z* + 1; (ii) *x* + 1/2, −*y* + 3/2, −*z* + 1; (iii) *x* − 1, *y* + 1/2, −*z* + 3/2, (iv) −*x* + 1, *y* − 1/2, *z* − 3/2.

Hydrogen bonds	D⋯A (Å)	H⋯A (Å)	D—H—A (°)	ρ_BCP_ (e Å^−3^)	*∇* ^2^ρ_BCP_ (e Å^−5^)	*H*_r_ (Ha a_0_^−3^)
O1A⋯H2A—O2A^i^	2.696 (2)	1.723	159.99	0.259	2.568	−0.034
O2A—H2A⋯O3A^ii^	2.696 (2)	1.723	159.99	0.255	2.551	−0.033
O1B⋯H2B—O2B^iii^	2.657 (1)	1.723	151.66	0.259	2.850	−0.035
O2B—H2B⋯O1B^iv^	2.657 (1)	1.723	151.66	0.255	2.838	−0.034

**Table:** 

Hydrogen bonds	S(D) (%)	S(H) (%)	S(A) (%)	S(D+H) (%)	S(A+H) (%)	S(D+H+A) (%)
O1A⋯H2A—O2A^i^	39.79	−9.14	23.86	30.65	14.72	54.51
O2A—H2A⋯O1A^ii^	39.84	−9.10	24.64	30.74	15.54	55.38
O1B⋯H2B—O2B^iii^	48.26	−9.32	33.52	38.94	24.20	72.46
O2B—H2B⋯O1B^iv^	46.51	−9.21	33.82	37.30	24.61	71.12

**Table:** (*a*) α-Ket

	Crystal			Pixel			CE		
	A⋯A	B⋯B	A⋯B	A⋯A	B⋯B	A⋯B	A⋯A	B⋯B	A⋯B
*E*_tot_	−33.57	−43.03	−33.68	−28.9	−36.6	−34.2	−30.0	−36.1	−42.9
*E*_coul_	–	–	–	−48.7	−59.3	−17.9	−43.2	−48.1	−14.0
*E*_pol_	–	–	–	−21.9	−28.1	−9.6	−8.7	−10.8	−3.9
*E*_energy-dispersive_	–	–	–	−9.7	−17.2	−58.1	−6.7	−12.8	−62.2
*E*_rep_	–	–	–	51.5	68.1	51.4	45.0	54.6	46.58

**Table:** (*b*) β-Ket

	Crystal		Pixel		CE	
	A⋯A	C—H⋯π	A⋯A	C—H⋯π	A⋯A	C—H⋯π
*E*_tot_	−91.86	−34.98	−62.2	−37.7	−71.4	−46.2
*E*_coul_	–	–	−152.1	−16.6	−133.3	−15.1
*E*_pol_	–	–	−78.0	−9.1	−29.0	−4.7
*E*_energy-dispersive_	–	–	−22.3	−56.1	−13.3	−62.8
*E*_rep_	–	–	190.2	44.1	166.1	45.3

**Table 7:** Lattice energies (kJ mol^−1^) for α-ket and β-ket major conformers

	α-Ket		β-Ket	
	Pixel	Crystal	Pixel	Crystal
*E*_tot_	−138.8	−155.93	−142.2	−164.60
*E*_coul_	−90.5	–	−112.4	–
*E*_pol_	−49.1	–	−63.1	–
*E*_energy-dispersive_	−159.3	–	−157.3	–
*E*_rep_	160.1	–	190.6	–
