# Supplementary material for: Crystal structure, interaction energies and experimental electron density of the popular drug ketoprophen
Source: IUCrJ. 2018 Oct 27;5(Pt 6):841–53. doi: 10.1107/S2052252518013222 (PMC6211533; doi:10.1107/S2052252518013222)
Supplement: Supplementary file 2 [file m-05-00841-sup2.pdf]

IUCrJ

Volume 5 (2018)

Supporting information for article:

Crystal structure, interaction energies and experimental electron density of the popular drug ketoprophen

Sylwia Pawlędzio, Anna Makal, Damian Trzybiński and Krzysztof Woźniak

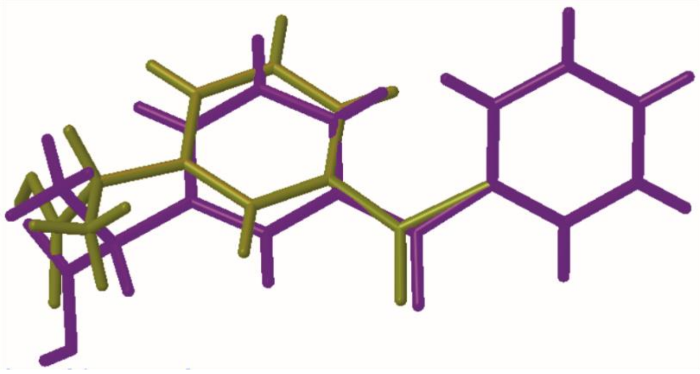

Figure S1 The molecular structure for **β-ket** with the disordered part – dark green colour represents the minor conformer.

a)

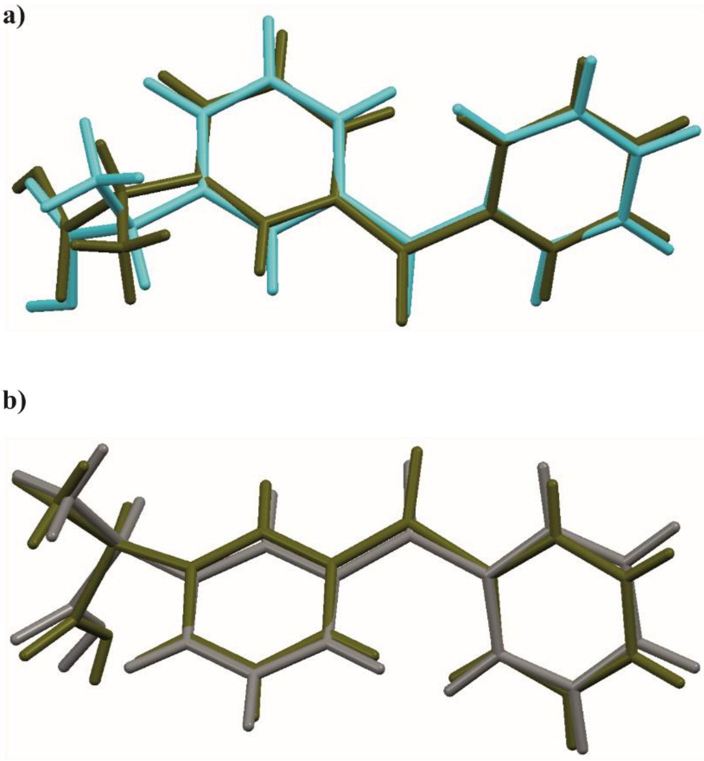

b)

Figure S2 The conformational differences visualized by superpositions of molecules: **α-ket** molecule A and **β-ket** minor conformer (a), **α-ket** molecule B and **β-ket** minor conformer (b). Coloring scheme: light blue – **α-ket** A molecule; gray – **α-ket** B molecule; dark green – **β-ket** minor conformer.

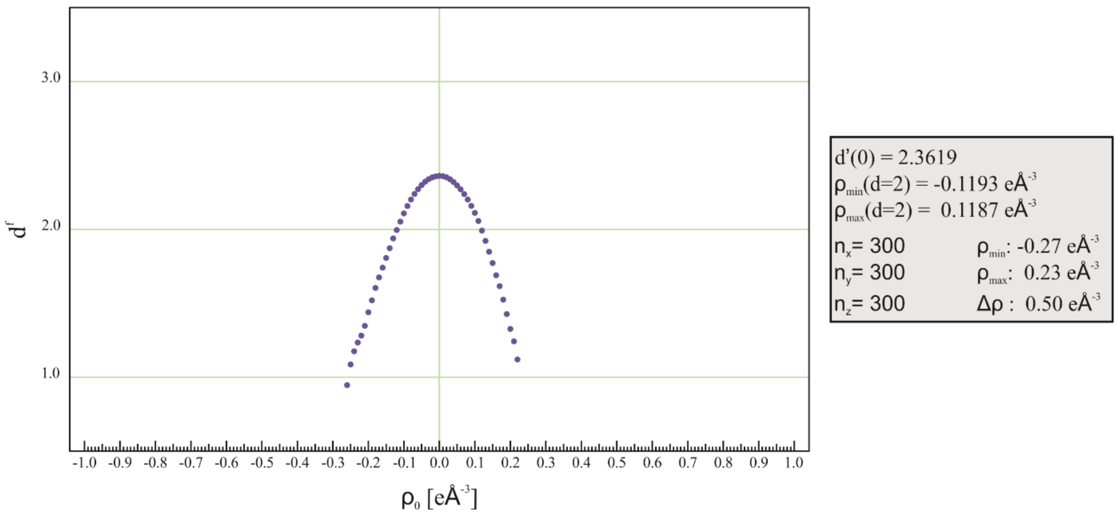

Figure S3 Fractal dimension vs. residual density after TAAM refinement for **α-ket**.

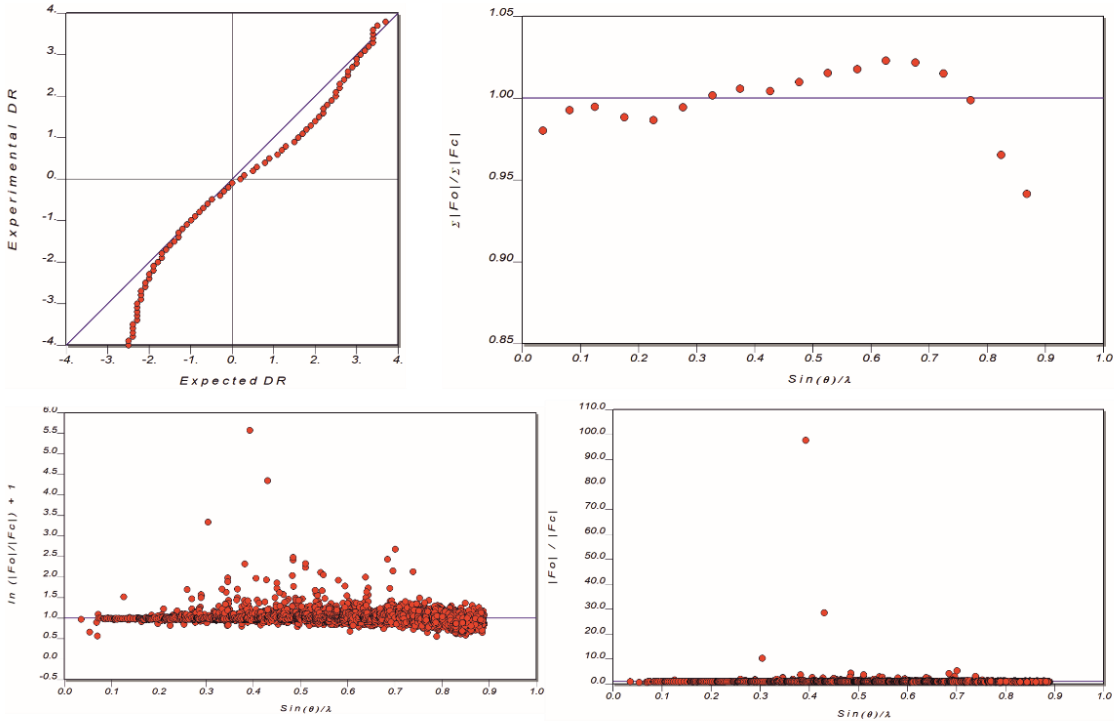

Figure S4 DRK and scale plots at 100 K data after TAAM refinement for **α-ket**.

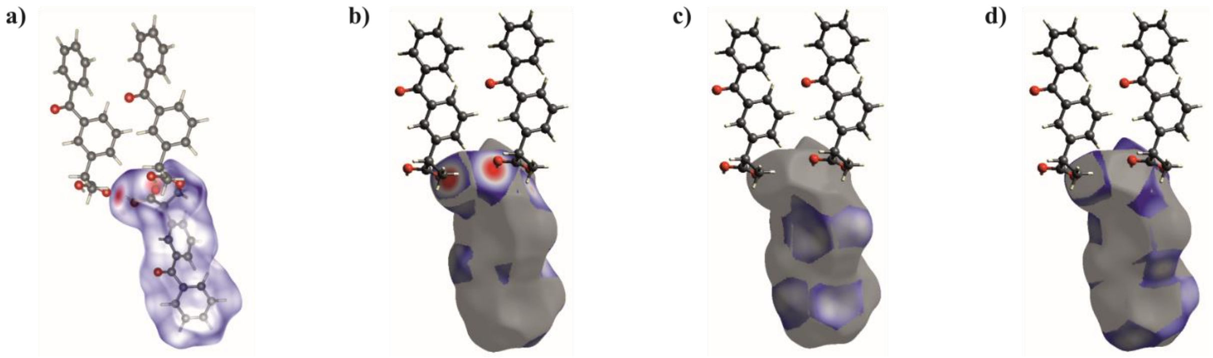

Figure S5 Hirshfeld surfaces for **α-ket** molecule A ((S)-enantiomer), a) all, b) O–H, c) C–H, d) H–H.

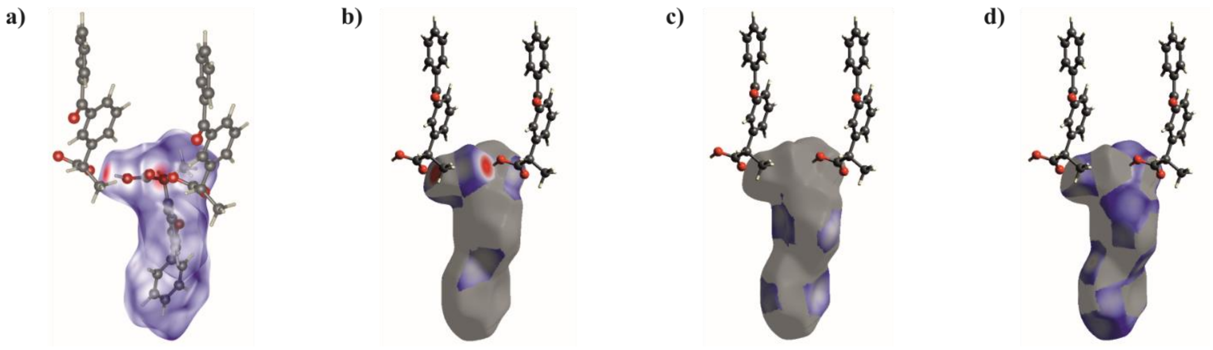

Figure S6 Hirshfeld surfaces for **α-ket** molecule B ((S)-enantiomer), a) all, b) O–H, c) C–H, d) H–H.

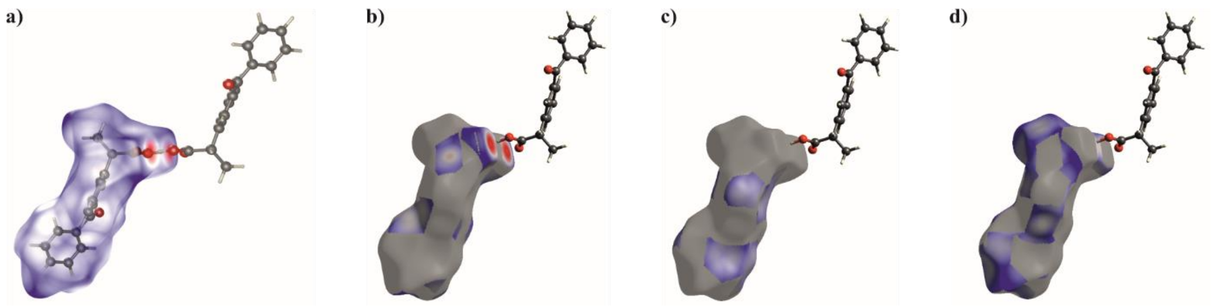

Figure S7 Hirshfeld surfaces for **β-ket** molecule main conformer ((RS)-enantiomer), a) all, b) O–H, c) C–H, d) H–H.

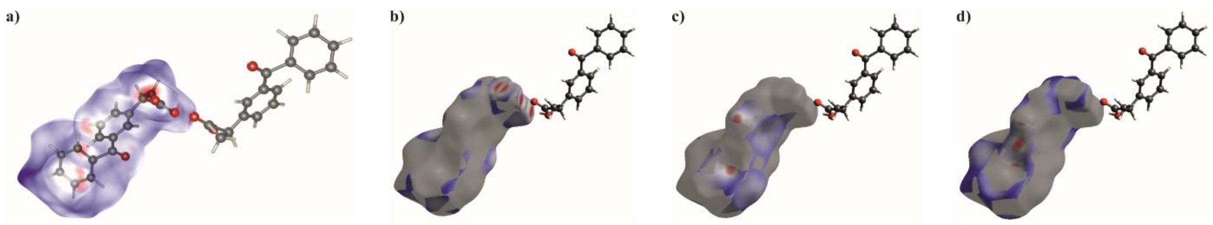

Figure S8 Hirshfeld surfaces for **β-ket** molecule minor conformer ((RS)-enantiomer), a) all, b) O–H, c) C–H, d) H–H.

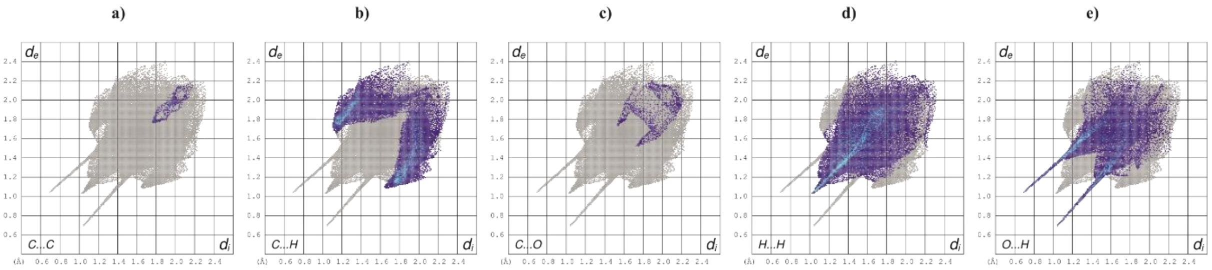

Figure S9 Fingerprint plot for **α-ket** molecule A ((S)-enantiomer).

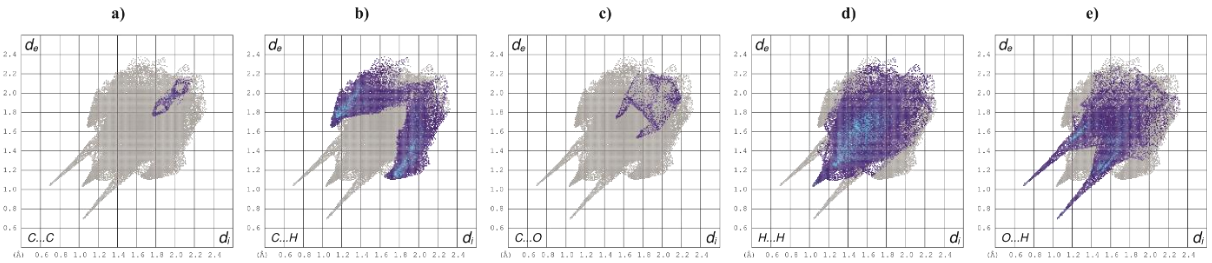

Figure S10 Fingerprint plot for **α-ket** molecule B ((S)-enantiomer).

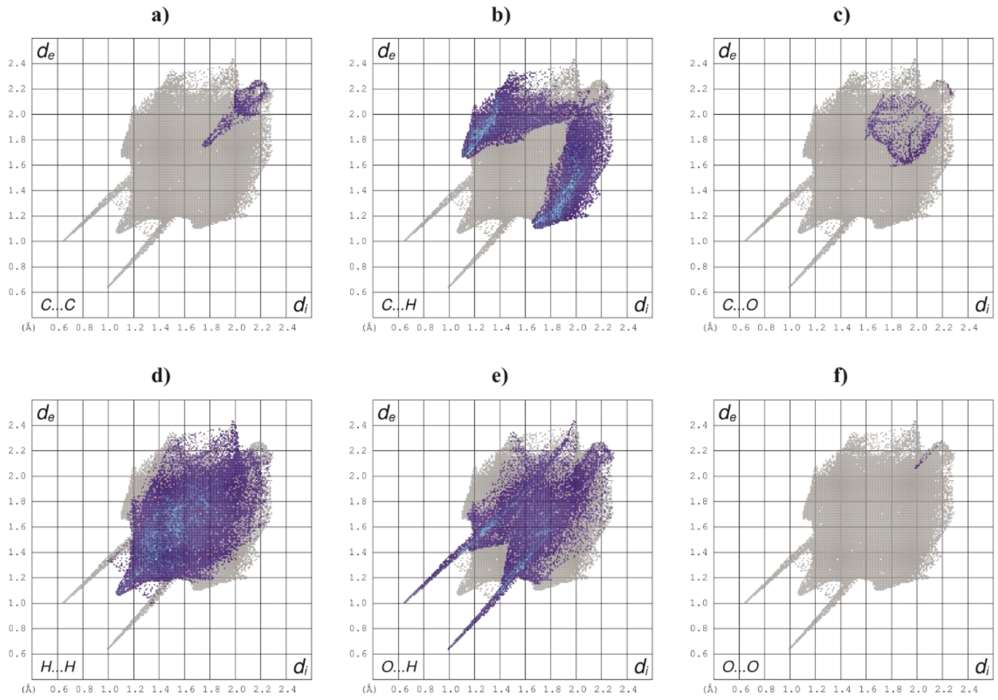

Figure S11 Fingerprint plot for **β-ket** molecule – main conformer ((RS)-enantiomer).

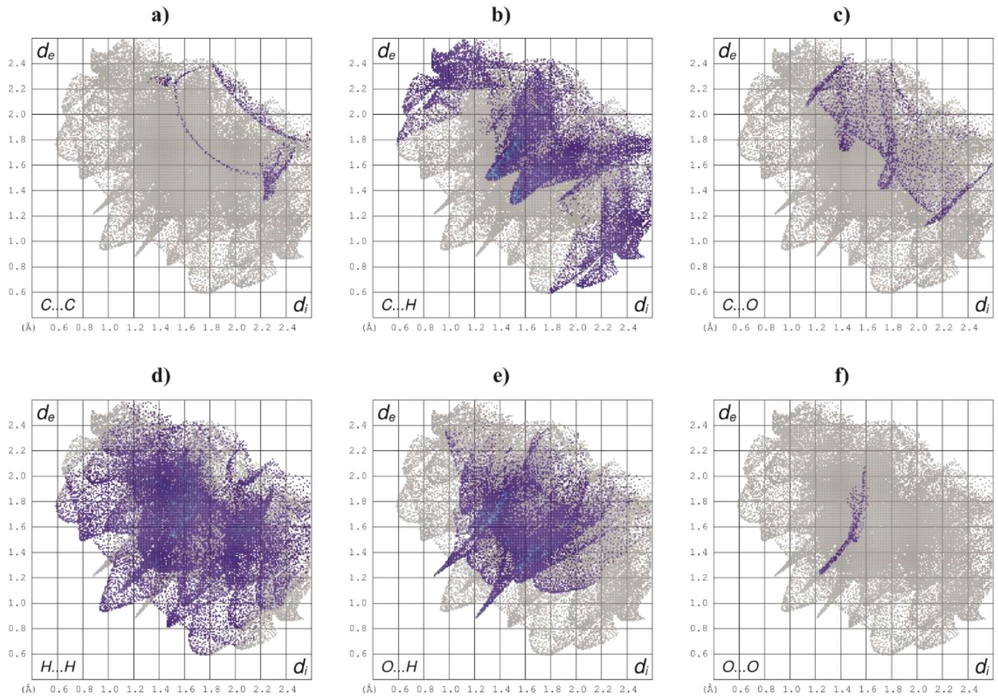

Figure S12 Fingerprint plots for **β-ket** molecule – minor conformer ((RS)-enantiomer).

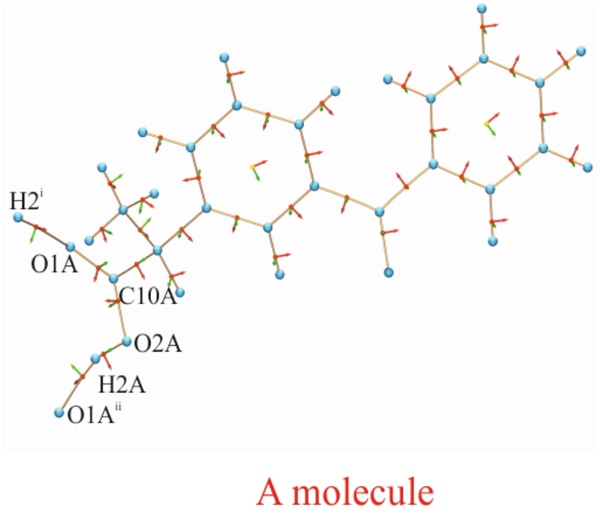

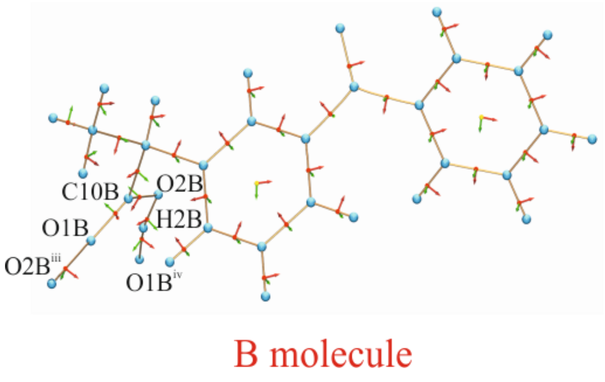

Figure S13 Molecular graphs for **α-ket**. Colour coding: bond path is shown as a golden path, RCP (3, +1) – as small yellow spheres, BCP (3, -1) – as small red spheres, NCP (3, -3) (atomic positions)

are shown as blue spheres. Symmetry codes: (i) x – 1/2, –y + 3/2, –z + 1; (ii) x + 1/2, –y + 3/2, –z + 1; (iii) x – 1, y + 1/2, –z + 3/2; (iv) –x + 1, y – 1/2, z – 3/2.

Table S1 A full list of BCP-s found for the **α-ket**.

Bond	ρ [eA^{-3}]	$\nabla^2\rho$ [eA^{-5}]	λ1	λ2	λ3	ε	G_r [Haa$_0^{-3}$]	V_r [Haa$_0^{-3}$]	H_r [Haa$_0^{-3}$]	G_r/ρ [Ha/e]	H_r/ρ [Ha/e]	$\lvert V_r\rvert/G_r$
O1A–C10A	2.857	-23.277	-25.80	-24.81	27.33	0.04	0.524	-1.289	-0.765	1.238	-1.808	2.461
O2A–H2A	2.17	-31.919	-31.8	-31.38	31.26	0.01	0.212	-0.756	-0.543	0.660	-1.690	3.560
O2A–C10A	2.203	-23.818	-18.42	-17.06	11.67	0.08	0.279	-0.806	-0.526	0.856	-1.613	2.884
O3A–C7A	2.826	-23.573	-24.61	-24.15	25.19	0.02	0.510	-1.264	-0.754	1.217	-1.801	2.480
C1A–C2A	2.11	-18.133	-15.58	-12.91	10.36	0.21	0.288	-0.764	-0.476	0.921	-1.522	2.653
C1A–C6A	2.077	-17.35	-15.27	-12.65	10.57	0.21	0.283	-0.745	-0.463	0.918	-1.503	2.637
C1A–C8A	1.636	-10.814	-11.11	-10.48	10.77	0.06	0.196	-0.504	-0.308	0.807	-1.270	2.573
C2A–H2AA	1.921	-21.023	-17.41	-17.02	13.40	0.02	0.208	-0.634	-0.426	0.731	-1.497	3.048
C2A–C3A	2.083	-17.381	-15.31	-12.68	10.60	0.21	0.284	-0.749	-0.465	0.921	-1.505	2.634
C3A–C4A	2.096	-17.697	-15.42	-12.78	10.50	0.21	0.286	-0.756	-0.470	0.922	-1.513	2.641
C3A–C7A	1.819	-14.049	-12.89	-11.80	10.64	0.09	0.226	-0.597	-0.371	0.837	-1.378	2.646
C4A–H4A	1.918	-20.981	-17.38	-16.98	13.38	0.02	0.207	-0.633	-0.425	0.730	-1.496	3.049
C4A–C5A	2.09	-17.691	-15.43	-12.87	10.60	0.20	0.284	-0.752	-0.468	0.918	-1.511	2.645
C5A–H5A	1.914	-20.883	-17.32	-16.93	13.37	0.02	0.207	-0.630	-0.424	0.729	-1.493	3.047
C5A–C6A	2.092	-17.746	-15.45	-12.88	10.59	0.20	0.285	-0.753	-0.469	0.918	-1.512	2.647
C6A–H6A	1.917	-20.945	-17.36	-16.97	13.38	0.02	0.207	-0.632	-0.425	0.730	-1.495	3.048
C7A–C11A	1.821	-14.107	-12.89	-11.84	10.63	0.09	0.226	-0.598	-0.372	0.837	-1.379	2.648
C8A–H8A	1.858	-19.293	-16.25	-16.22	13.19	0.00	0.201	-0.602	-0.401	0.730	-1.457	2.996
C8A–C10A	1.767	-13.787	-12.53	-11.62	10.36	0.08	0.212	-0.567	-0.355	0.810	-1.356	2.674
C8A–C9A	1.590	-10.749	-10.73	-10.71	10.68	0.00	0.184	-0.479	-0.295	0.779	-1.252	2.608
C9A–H9AA	1.906	-21.09	-17.04	-16.93	12.89	0.01	0.203	-0.625	-0.422	0.719	-1.493	3.078
C9A–H9AB	1.903	-20.988	-16.99	-16.89	12.90	0.01	0.203	-0.623	-0.421	0.719	-1.491	3.074
C9A–H9AC	1.903	-20.994	-17.00	-16.89	12.90	0.01	0.203	-0.623	-0.421	0.719	-1.491	3.074
C11A–C12A	2.08	-17.339	-15.28	-12.66	10.60	0.21	0.284	-0.747	-0.463	0.920	-1.504	2.634
C11A–C16A	2.087	-17.489	-15.34	-12.71	10.56	0.21	0.285	-0.751	-0.466	0.921	-1.508	2.637
C12A–H12A	1.919	-21.01	-17.39	-16.99	13.37	0.02	0.208	-0.633	-0.425	0.730	-1.496	3.050
C12A–C13A	2.094	-17.77	-15.46	-12.89	10.58	0.20	0.285	-0.755	-0.470	0.919	-1.513	2.646
C13A–H13A	1.924	-21.152	-17.45	-17.05	13.34	0.02	0.208	-0.636	-0.428	0.730	-1.499	3.055
C13A–C14A	2.089	-17.677	-15.42	-12.86	10.60	0.20	0.284	-0.752	-0.468	0.918	-1.510	2.645

C14A–H14A	1.927	-21.239	-17.49	-17.09	13.34	0.02	0.208	-0.637	-0.429	0.730	-1.501	3.057
C14A–C15A	2.092	-17.744	-15.44	-12.88	10.58	0.20	0.285	-0.753	-0.469	0.918	-1.512	2.647
C15A–H15A	1.912	-20.842	-17.30	-16.91	13.37	0.02	0.207	-0.629	-0.423	0.729	-1.492	3.047
C15A–C16A	2.097	-17.843	-15.49	-12.92	10.56	0.20	0.286	-0.756	-0.471	0.919	-1.515	2.648
C16A–H16A	1.918	-20.951	-17.36	-16.97	13.38	0.02	0.208	-0.633	-0.425	0.731	-1.495	3.047
O1B–C10B	2.853	-23.619	-25.72	-24.73	26.83	0.04	0.520	-1.285	-0.765	1.230	-1.809	2.471
O2B–H2B	2.172	-32.085	-31.90	-31.48	31.29	0.01	0.212	-0.756	-0.545	0.658	-1.692	3.571
O2B–C10B	2.197	-23.969	-18.34	-16.95	11.33	0.08	0.276	-0.801	-0.525	0.849	-1.612	2.900
O3B–C7B	2.820	-24.026	-24.50	-24.03	24.51	0.02	0.504	-1.257	-0.753	1.206	-1.803	2.495
C1B–C2B	2.110	-18.146	-15.59	-12.91	10.35	0.21	0.288	-0.764	-0.476	0.920	-1.522	2.654
C1B–C6B	2.077	-17.358	-15.27	-12.65	10.57	0.21	0.283	-0.745	-0.463	0.918	-1.503	2.637
C1B–C8B	1.631	-10.722	-11.03	-10.47	10.78	0.05	0.195	-0.501	-0.306	0.806	-1.267	2.571
C2B–H2BA	1.915	-20.843	-17.32	-16.93	13.41	0.02	0.207	-0.631	-0.424	0.731	-1.493	3.042
C2B–C3B	2.072	-17.15	-15.22	-12.60	10.66	0.21	0.282	-0.743	-0.460	0.920	-1.499	2.630
C3B–C4B	2.092	-17.611	-15.38	-12.75	10.52	0.21	0.286	-0.754	-0.468	0.921	-1.511	2.640
C4B–C7B	1.811	-13.906	-12.83	-11.73	10.65	0.09	0.224	-0.593	-0.368	0.835	-1.373	2.643
C4B–H4B	1.912	-20.813	-17.3	-16.9	13.39	0.02	0.207	-0.629	-0.423	0.730	-1.492	3.044
C4B–C5B	2.076	-17.367	-15.3	-12.76	10.69	0.20	0.282	-0.744	-0.462	0.917	-1.503	2.639
C5B–H5B	1.917	-20.987	-17.37	-16.97	13.35	0.02	0.207	-0.632	-0.425	0.729	-1.495	3.051
C5B–C6B	2.087	-17.628	-15.4	-12.84	10.62	0.20	0.284	-0.751	-0.467	0.918	-1.509	2.644
C6B–H6B	1.923	-21.087	-17.42	-17.03	13.37	0.02	0.208	-0.635	-0.427	0.731	-1.498	3.051
C7B–C11B	1.815	-13.992	-12.84	-11.8	10.64	0.09	0.225	-0.595	-0.370	0.836	-1.375	2.646
C8B–H8B	1.861	-19.365	-16.28	-16.26	13.18	0.00	0.201	-0.604	-0.402	0.730	-1.458	2.998
C8B–C10B	1.766	-13.813	-12.55	-11.62	10.36	0.08	0.212	-0.567	-0.355	0.809	-1.356	2.677
C8B–C9B	1.592	-10.787	-10.74	-10.73	10.68	0.00	0.184	-0.480	-0.296	0.779	-1.254	2.609
C9B–H9BA	1.897	-20.817	-16.92	-16.81	12.91	0.01	0.202	-0.620	-0.418	0.719	-1.487	3.068
C9B–H9BB	1.904	-21.023	-17.01	-16.9	12.89	0.01	0.203	-0.624	-0.421	0.719	-1.492	3.075
C9B–H9BC	1.903	-20.985	-16.99	-16.89	12.89	0.01	0.203	-0.623	-0.421	0.719	-1.491	3.073
C11B–C12B	2.082	-17.377	-15.3	-12.68	10.59	0.21	0.284	-0.748	-0.464	0.921	-1.505	2.635
C11B–C16B	2.095	-17.678	-15.41	-12.77	10.5	0.21	0.286	-0.756	-0.470	0.922	-1.512	2.641
C12B–H12B	1.914	-20.843	-17.31	-16.92	13.39	0.02	0.207	-0.631	-0.423	0.730	-1.493	3.044
C12B–C13B	2.102	-17.987	-15.54	-12.96	10.52	0.20	0.286	-0.759	-0.473	0.919	-1.518	2.652
C13B–H13B	1.92	-21.065	-17.4	-17.01	13.35	0.02	0.207	-0.633	-0.426	0.729	-1.497	3.053
C13B–C14B	2.068	-17.188	-15.23	-12.7	10.73	0.20	0.281	-0.740	-0.459	0.916	-1.498	2.635
C14B–H14B	1.922	-21.123	-17.43	-17.03	13.33	0.02	0.208	-0.634	-0.427	0.729	-1.499	3.055

C14B–C15B	2.083	-17.546	-15.37	-12.81	10.63	0.20	0.283	-0.748	-0.465	0.917	-1.507	2.643
C15B–H15B	1.923	-21.154	-17.44	-17.05	13.34	0.02	0.208	-0.635	-0.427	0.729	-1.499	3.056
C15B–C16B	2.08	-17.441	-15.33	-12.78	10.67	0.20	0.283	-0.747	-0.464	0.918	-1.505	2.640
C16B–H16B	1.92	-21.008	-17.39	-16.99	13.38	0.02	0.208	-0.634	-0.426	0.731	-1.497	3.049
H2B ··O1B(X4)	0.255	2.838	-1.52	-1.37	5.73	0.11	0.032	-0.034	-0.002	0.843	-0.063	1.075
O1B ··H2B(X4)	0.259	2.850	-1.54	-1.38	5.78	0.11	0.032	-0.035	-0.003	0.840	-0.070	1.083
O1A ··H2A(X3)	0.259	2.568	-1.56	-1.48	5.6	0.05	0.030	-0.034	-0.004	0.789	-0.095	1.120
H2A ··O1A(X3)	0.255	2.551	-1.54	-1.46	5.55	0.05	0.030	-0.033	-0.003	0.790	-0.090	1.113

Table S2 Net atomic charges for **α-ket**.

Net atomic charges [eÅ^{-3}]									
Atom	Multipole	Stockholder	QTAIM	Crystal	Atom	Multipole	Stockholder	QTAIM	Crystal
O1A	-0.0777	-0.2069	-1.0972	-0.4918	O1B	-0.0777	-0.2182	-1.089	-0.4932
O2A	-0.1321	-0.1344	-0.9937	-0.4818	O2B	-0.1321	-0.1377	-0.993	-0.4803
H2A	0.1892	0.1793	0.5314	0.3651	H2B	0.1892	0.1794	0.5315	0.3801
O3A	-0.0525	-0.1721	-0.9751	-0.4503	O3B	-0.0525	-0.1719	-0.9711	-0.4462
C1A	-0.0539	-0.0062	-0.0089	0.0798	C1B	-0.0539	-0.0045	-0.0074	0.0941
C2A	0.0777	-0.0596	-0.0189	-0.1369	C2B	0.0777	-0.0587	-0.019	-0.1385
H2AA	-0.0638	0.0347	0.0043	0.1252	H2BA	-0.0638	0.0349	0.0045	0.1251
C3A	-0.0539	-0.0544	-0.0467	0.0328	C3B	-0.0539	-0.0545	-0.0469	0.0328
C4A	0.0777	-0.0448	-0.006	-0.1035	C4B	0.0777	-0.0457	-0.0066	-0.1010
H4A	-0.0638	0.0389	0.0027	0.1166	H4B	-0.0638	0.0389	0.0042	0.1176
C5A	0.0777	-0.0334	0.0107	-0.0959	C5B	0.0777	-0.0335	0.0106	-0.0897
H5A	-0.0638	0.046	0.0046	0.1139	H5B	-0.0638	0.0461	0.0039	0.1137
C6A	0.0777	-0.0432	-0.0052	-0.1190	C6B	0.0777	-0.0455	-0.0086	-0.1140
H6A	-0.0638	0.0373	0.0031	0.1147	H6B	-0.0638	0.0366	0.0047	0.0929
C7A	-0.1050	0.1120	0.8673	0.3012	C7B	-0.1050	0.1123	0.8651	0.3062
C8A	0.2046	0.0111	0.1424	-0.1895	C8B	0.2046	0.0118	0.1222	-0.1910

H8A	-0.0784	0.0292	-0.0381	0.1320	H8B	-0.0784	0.0299	-0.0383	0.1369
C9A	0.4268	-0.0366	0.171	-0.3367	C9B	0.4268	-0.0384	0.1653	-0.3489
H9AA	-0.0943	0.0464	-0.0107	0.1277	H9BA	-0.0943	0.0453	-0.0042	0.1327
H9AB	-0.0943	0.0479	-0.0124	0.1221	H9BB	-0.0943	0.0470	-0.0118	0.1198
H9AC	-0.0943	0.0481	-0.0122	0.1213	H9BC	-0.0943	0.0488	-0.0117	0.1192
C10A	-0.0564	0.2045	1.5254	0.5852	C10B	-0.0564	0.2076	1.4999	0.5741
C11A	-0.0539	-0.0523	-0.0536	0.0223	C11B	-0.0539	-0.0521	-0.0523	0.0221
C12A	0.0777	-0.0505	-0.0039	-0.1015	C12B	0.0777	-0.0504	-0.0084	-0.0956
H12A	-0.0638	0.0355	0.0038	0.1186	H12B	-0.0638	0.0353	0.0022	0.1196
C13B	0.0777	-0.033	0.0079	-0.1021	C13B	0.0777	-0.034	0.0058	-0.1146
H13B	-0.0638	0.0463	0.0042	0.1031	H13B	-0.0638	0.044	0.005	0.0872
C14A	0.0777	-0.0322	0.0077	-0.0963	C14B	0.0777	-0.0343	0.0107	-0.0970
H14A	-0.0638	0.0463	0.0039	0.0746	H14B	-0.0638	0.0381	0.0069	0.0936
C15A	0.0777	-0.033	0.0073	-0.1108	C15B	0.0777	-0.0332	0.0088	-0.1056
H15A	-0.0638	0.0461	0.0047	0.1115	H15B	-0.0638	0.0461	0.0038	0.1074
C16A	0.0777	-0.0448	-0.008	-0.0865	C16B	0.0777	-0.0455	-0.0076	-0.0846
H16A	-0.0638	0.04	0.0032	0.1291	H16B	-0.0638	0.0406	0.0035	0.1305

Table S3. Dimer interaction and lattice crystal energies for **β-ket** minor.

Energy kJ/mol	β-ket minor							
	Dimer						Lattice	
	Crystal		Pixel		CE		Crystal	Pixel
	A···A	C–H···π	A···A	C–H···π	A···A	C–H···π		
**E_{tot}**	-89.3	-32.8	-68.1	-30.1	-72.0	-37.4	-147.69	-128.6
**E_{coul}**			-121.4	-8.6	-111.5	-17.8		-99.3
**E_{pol}**			-55.9	-5.7	-24.5	-5.9		-53.1
**E_{disp}**			-20.4	-43.6	-12.4	-57.3		-158.8
**E_{rep}**			129.6	27.8	121.1	57.7		182.6

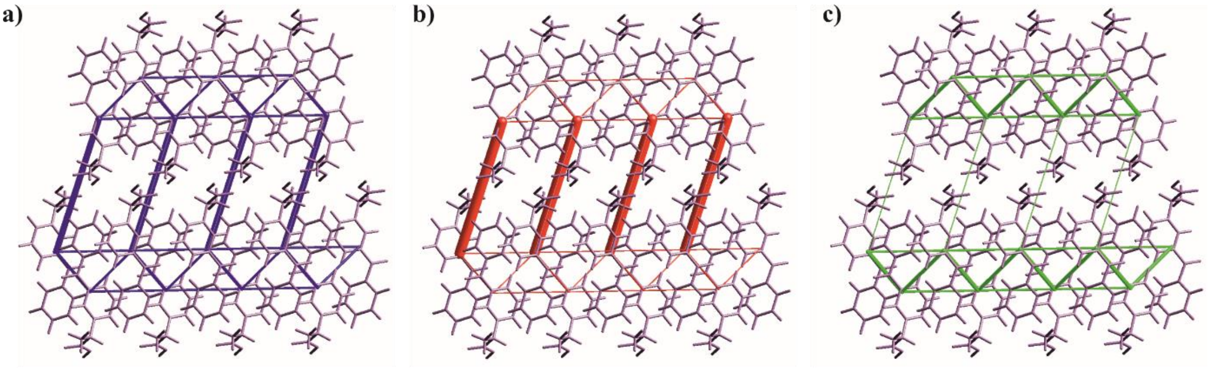

Figure S14 Energy frameworks for the crystal structure of **β-ket** minor conformer (a-c). Tube size is set to the 36, cut-off to 8 kJ/mol. Colouring scheme: total energy – blue, electrostatic energy – red and dispersion energy – green.
